# Population Explosions of Tiger Moth Lead to Lepidopterism Mimicking Infectious Fever Outbreaks

**DOI:** 10.1371/journal.pone.0152787

**Published:** 2016-04-13

**Authors:** Pallara Janardhanan Wills, Mohan Anjana, Mohan Nitin, Raghuveeran Varun, Parayil Sachidanandan, Tharaniyil Mani Jacob, Madhavan Lilly, Raghava Varman Thampan, Koyikkal Karthikeya Varma

**Affiliations:** 1 MIMS Research Foundation, Malabar Institute of Medical Sciences (Aster MIMS), Kozhikode, Kerala, India; 2 ICFO-Institut de Ciències Fotòniques, Barcelona, Spain; 3 State Forensic Science Laboratory, Thiruvananthapuram, Kerala, India; 4 Department of Statistics, Nirmala College, Muvattupuzha, Kerala, India; Charles University in Prague, CZECH REPUBLIC

## Abstract

Lepidopterism is a disease caused by the urticating scales and toxic fluids of adult moths, butterflies or its caterpillars. The resulting cutaneous eruptions and systemic problems progress to clinical complications sometimes leading to death. High incidence of fever epidemics were associated with massive outbreaks of tiger moth *Asota caricae* adult populations during monsoon in Kerala, India. A significant number of monsoon related fever characteristic to lepidopterism was erroneously treated as infectious fevers due to lookalike symptoms. To diagnose tiger moth lepidopterism, we conducted immunoblots for tiger moth specific IgE in fever patients’ sera. We selected a cohort of patients (n = 155) with hallmark symptoms of infectious fevers but were tested negative to infectious fevers. In these cases, the total IgE was elevated and was detected positive (78.6%) for tiger moth specific IgE allergens. Chemical characterization of caterpillar and adult moth fluids was performed by HPLC and GC-MS analysis and structural identification of moth scales was performed by SEM analysis. The body fluids and chitinous scales were found to be highly toxic and inflammatory in nature. To replicate the disease in experimental model, wistar rats were exposed to live tiger moths in a dose dependant manner and observed similar clinico-pathological complications reported during the fever epidemics. Further, to link larval abundance and fever epidemics we conducted cointegration test for the period 2009 to 2012 and physical presence of the tiger moths were found to be cointegrated with fever epidemics. In conclusion, our experiments demonstrate that inhalation of aerosols containing tiger moth fluids, scales and hairs cause systemic reactions that can be fatal to human. All these evidences points to the possible involvement of tiger moth disease as a major cause to the massive and fatal fever epidemics observed in Kerala.

## Introduction

Recent widespread and recurrent fever epidemics that occurred in tropical countries including India have experienced high morbidity and mortality [1, 2]. Kerala, the south-western state of India faced disastrous fever outbreaks, where more than 23.4 million people (S1 Fig) [3] were affected leading to approximately 2,250 deaths since the year 2006. Despite having aggressive public health-care practices, recent occurrences of epidemic pose a great challenge to the extensive health programme in the State. The advent of fever epidemics ravaged all the fourteen districts of the State and has drawn worldwide attention due to its explosive nature [1, 4–7]. In the midst of epidemics, clinicians in our hospital encountered a plethora of weird symptoms and were puzzled as the fever showed hallmark symptoms of chikungunya and at times of dengue. The tests, however, were negative in a number of cases.

Multitude of unusual symptoms consisting cutaneous signs like maculopapular or petechial rash, soft tissue necrosis with extensive sloughing and hyperpigmentation to systemic complaints including fever with chills, headache, nausea, vomiting and diarrhea, sore throat, ecchymosis, large hives, edema, crippling arthralgia and arthritis, conjunctivitis, pharyngitis, respiratory distress, decrease in platelet counts, multi-organ disorders and reduced electrolyte levels have been observed (S1 Table) [4, 5, 7]. More seriously, incidence of meconium stained amniotic fluid and meconium aspiration syndrome associated with fever was observed in pregnant victims [6].

To our surprise, symptoms of different infectious diseases originate simultaneously during the epidemics and disappear altogether during decline phase of the epidemics. Further, a significant number of cases were diagnosed negative for almost all known infectious fever diseases (S2 Table), and were classified as suspected cases (S1 Fig) [3], leading to the assumption that the diseases are emanating from a unique source. Here, we investigate the involvement of tiger moth *Asota caricae* Fabricius (Lepidoptera: Noctuidae: Aganainae) induced toxic-allergic disease in fever patients during monsoon that mimics the symptoms of infectious disease. With the onset of monsoon, millions of tiger moth caterpillars infest host plants in the region and disease erupts during the eclosion of adult moths. On the other hand, fever outbreaks decline when the cyclic event moves into the larval and pupa stages. Adult moths are strongly attracted to artificial night lights and once entered in human habitation, they circle around bright lights, extricate scales, hairs and/or release deadly chemicals into the ambient and inhalation of these blends leads to severe forms of fever syndrome in human. In most instances, these symptoms mimic that of infectious fever outbreaks. In general, the pathophysiologic manifestations of toxic moths and/or caterpillar exposures are classified into distinct clinical syndromes that include erucism (reactions due to caterpillars), lepidopterism (cutaneous and systemic signs), pararamose (severe arthralgia and arthritis due to *Premolis* caterpillars), lonomism (contact with *Lonomia* caterpillars), dendrolomiasis (exposure to *Dendrolimus* caterpillars) and ophthalmia nodosa (ocular involvement) [8–15]. Several other unusual clinical presentations were also reported during the epidemic and one of them was meconium stained amniotic fluid and associated meconium aspiration syndrome in pregnant victims [6]. These conditions have a close similarity with equine fetal loss following exposure to eastern tent caterpillars and processionary caterpillars [16, 17]. Prevalence of tiger moth lepidopterism in India is not well documented and we suggest that heavy infestations of tiger moth can facilitate huge epidemics that lead to adverse reactions of lepidopterism or other related syndromes.

In this study, we conducted the chemical characterization of tiger moth fluids which are responsible for the immuno-inflammatory response in humans. Further, we detected tiger moth specific IgE antibody in fever patients’ sera and estimated the release of proinflammatory cytokine TNF-α in the associated joint disease. In addition, we replicated tiger moth disease in a rat model based on the clinico-pathological complications observed in fever patients with joint ankylosis. Finally, we cointegrated field estimates of tiger moth caterpillar abundance with incidence of epidemics and with meteorological conditions which are considered to be the factors behind the explosion of tiger moths.

## Materials and Methods

### Patients’ Survey

Massive fever epidemics occurred in Kerala in the year 2007 and witnessed unusual clinico-pathological complications among fever affected patients where most persisting was the fever with joint pain. We conducted a follow-up household survey in the year 2008 among these patients by giving an initial oral description about the fever epidemics and obtained consent for participating in the survey. Each patient was requested to fill a questionnaire with their name and address, age, weight, symptoms of the disease, disabilities during convalescence, prolonged effect of the disease, treatment taken and their perception about the cause of the disease.

### Moth Census, Fever Data and Environmental Data

**Fig 1:**
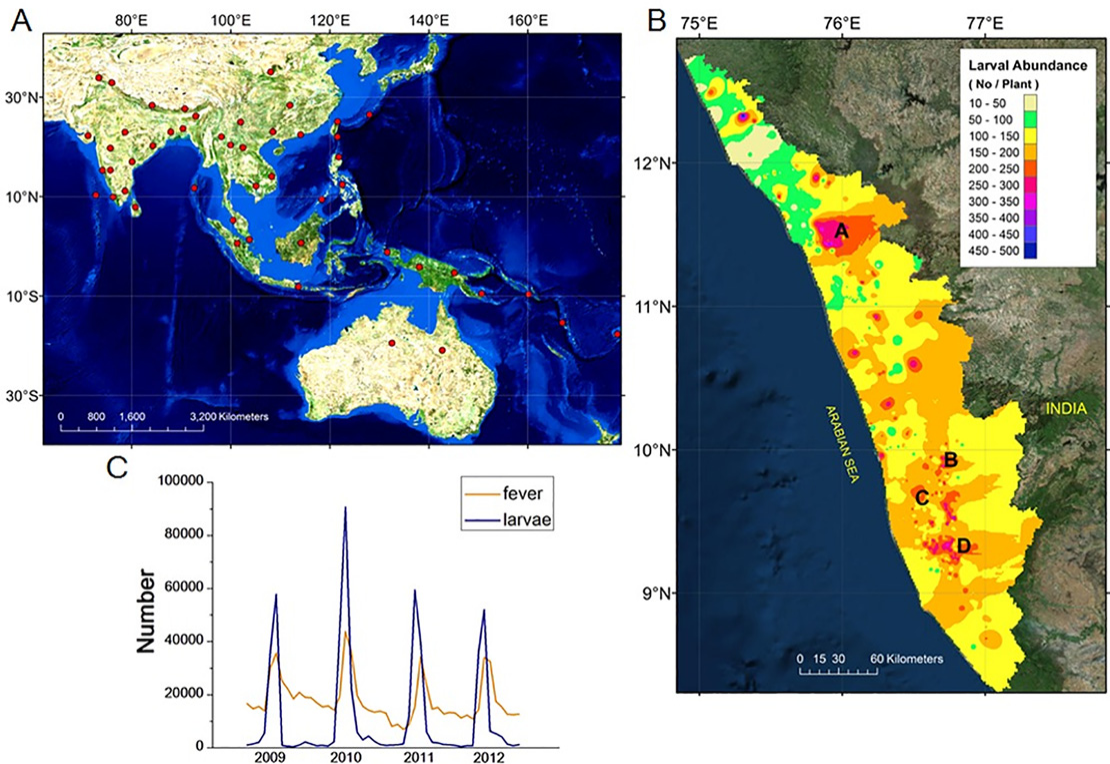
Tiger moth habitats and cointegration of larval versus epidemic waves. (A) Geographical range of *A*. *caricae* (marked as red) includes Indo-Australian tropics to Queensland and Vanuatu (Ref. 18 & 48). (B) Abundance of tiger moth caterpillars infested on *F*. *hispida* in Kerala during the year 2009. (C). The mean larval abundance cointegrate with total number of fever cases in selected districts of Kerala (marked as A-D) from the year 2009 to 2012. All the regions from hilly terrains to low lying lands of Kerala are heavily distributed with host plants that have close proximity to houses with illuminated lights. The maps were created using ArcGIS 10 software.

We conducted a survey in Kerala (8° 36’ to 12° 30’ N and 74° 59’ to 77° 03’ E, altitude up to 770 m above sea level) during July to September 2009 to understand the occurrence, geographical distributions and population sizes of tiger moth in the State. For that we collected data on the number of pioneer figs infested with tiger moth caterpillars and the number of caterpillars per plant from randomly positioned three transects (200 x 2 m) at 10 km intervals. Host plants were represented by three tier habits herbs, shrubs and trees in the region and the abundance of caterpillars was measured on herbs, shrubs and trees separately. In this study, we considered main branches as shoot in herbs and side branches on the main stem as shoots in shrubs and trees. We observed that caterpillars are equally distributed on each shoot of the plant and size of the population was measured in terms of caterpillars per shoot (measured on randomly selected 5 to 25 shoots depending on the size of the plant) and then extrapolated to per plant and finally to the study area. No specific permissions were required for conducting these activities at different locations in Kerala because the invasive host plants were available on both sides of the public roads including national highways, state highways and even small pocket roads. The larvae forage on the leaves when atmospheric temperature drops, so the censuses were carried out in the early morning (between 05:00 and 08:00 h) and late evening hours (18:00 to 24:00 h) and the sampling extended to 06:00 to 11:00 h and 14:00 to 22:00 h on rainy days. We selected four districts Kozhikode, Idukki, Kottayam and Pathanamthitta (represent major stations A-D respectively in Fig 1B) where huge fever outbreaks reported in the year 2007 to study the moth population characteristics and its relation with fever outbreaks. At each station we selected substations (A-9, B-15, C-12 and D-15 substations) and from each substation three transects (each transect 200 x 2 m) at 10 km intervals were marked and data on number of host plants, egg masses, caterpillars, pupae and parasitation were recorded monthly from 2009 to 2012. Size of the population was measured in terms of caterpillars per shoot and then extrapolated to per plant and finally to the study area. The observed larval mortality (due to parasitation and heavy rainfall) was deducted from larval abundance values. The mortality at pupal stages was not considered as it was observed to be very negligible (3.6%, *n* = 852). Geographic positions of the study area (S1 Dataset) were marked using GPSMAP 76CSx, Garmin and the dataset was exported to geographical information software (ArcGIS 10) to display geographical distributions and population sizes of the moth. Meteorological data on relative humidity, temperature, and rainfall were obtained from Indian Space Research Organization, Centre for Water Resources Development and Management and India Meteorological Department, India. Monthly fever data was collected from State Disease Control and Monitoring Cell and Directorate of Health Services (DHS), Kerala, India. Source of daily fever death reports were taken from DHS website and *Malayala Manorama* online edition. Geographical range of *A*. *caricae* habitats includes Asia to Pacific Islands and the positions were marked using ArcGIS 10 software.

### Moth Rearing

Egg masses, larvae and pupae were collected from the field and reared in the laboratory under controlled conditions. We further confirm that the field studies did not involve any endangered or protected plant or animal species. Body fluids collected separately from male and female moths in sterile containers immediately after the emergence and stored at –20°C until use. Secretions from fourth, fifth and late fifth instars were collected from the prothorax region and stored immediately at –20°C until use. Mating experiments were conducted in special containers supplied with diluted honey in water (1:1) as food. Precautionary steps were taken while handling adult moths using protective cloths, gloves and special face masks.

### HPLC and GC-MS Analysis

High performance liquid chromatography of moth fluids was performed and the purified fraction was subjected to Gas Chromatograph-Mass Spectrometry (GC-MS) analysis. The HPLC was Konikrom LC system with UV-VIS and Fluorescence Detectors (Konik HPLC, Konikrom). Separation was carried out at ambient temperature using Vydac C_18_ column (250 mm x 4.6 mm). The analysis was performed by gradient elution at a flow rate of 1.1 mL/min. The mobile phase composition consisting of Buffer A: 95% Water + 5% Acetonitrile (Merck), Buffer B: Acetonitrile containing 0.01% TFA (Sigma-Aldrich). The detector was set at 280 and 248 nm and peak areas were integrated automatically using Konikrom Plus software programme. For GC-MS, Konik HRGC gas chromatograph linked to MS system equipped with a fused silica column (25 m x 0.25 mm i.d) was used (Konik HRGC-4000 with MS Q12 detector, Konikrom). The temperature programming was 50°C for 2 min, 125°C@15°C/min, continued for 2 min, and 10°C/min up to 275°C for 5 min. Ion source temperature and interface temperature were 225°C, and 150°C respectively. Standard comparison of chemical components was done with NIST Mass Spectral Library Version 2.0.

Histamines and imidazole in tiger moths and caterpillar secretions were quantified by HPLC using standard chemicals (Sigma-Aldrich). Separation was carried out using C_18_ column (150 mm x 4.5 mm x 5*μ*m). The injection volume was 10 *μ*L and the analysis was performed by gradient elution at a flow rate of 1.5 mL/min. The gradient mobile phase consisting of aqueous solution of ammonium dihydrogen phosphate (0.05 M) and the final composition of ammonium dihydrogen phosphate was 0.0075 M. The column oven temperature was 25°C. The detector was set at 230 nm and peak areas were integrated (1260 Infinity Series, Agilent).

Volatiles in caterpillar and moth secretions were analyzed by GC-MS analysis. Briefly, 10 *μ*L of moth fluid was reconstituted with acetonitrile (1:1 v/v) and 1.0 *μ*L of the sample was injected directly into the GC (7890A, Agilent) linked to MS system (5975C Inert XL MSD, Agilent) equipped with columns DB-5 and DB-5ms, 30 m x 0.25 mm x 0.25 *μ*m. The temperature programming was 80°C for 5 min, 250°C@25°C/min, continued for 2 min. The injector temperature was 240°C and the detector temperature was 250°C respectively. The flow rate was 1.0 *μ*L/min. Standard comparison of volatiles was done with NIST Mass Spectral Library Version 2.0. The octan-1-ol in caterpillar and moth secretions was quantified using standard octan-1-ol (Merck). Thymoquinone standard was procured from Sigma-Aldrich.

### SEM Analysis

Scales from different parts of the moth (2 females and 2 males) were visualized under SEM (Hitachi SU6600). Prior to imaging, the samples were sputtered with gold (Hitachi, E1010).

### Peptide Analysis

Peptide mass fingerprinting was performed on MALDI-TOF-MS (UltrafleXtreme, Bruker Daltonics). Moth secretions were dissolved in a mixture of deionized water containing 5% ACN. α-cyano-4-hydroxycinnamic acid (0.1 mmol/L) was used as the matrix. The samples were sandwiched in the matrix on the MALDI plate using ZIP-Tip and exposed to laser source (power 70–80) and the peaks were detected using pre-calibrated detector (calibrated using angiotensin, insulin and BSA).

### Polyacrylamide Gel Electrophoresis of Tiger Moth Fluids

Moth fluids collected were mixed with lysis buffer (TEMP buffer consisting of 10 mM Tris-HCl (pH 7.6), 1 mM EDTA, 12 mM monothioglycerol and 0.2 mM phenylmethanesulfonyl fluoride) and resolved on 15% SDS gel (Mini-Protean Tetra Cell, BioRad), stained with Coomassie Brilliant Blue and photographed (Gel Doc EZ Imager, BioRad).

### Estimation of Total IgE in Patients’ Sera

Clinical study was approved by the Institutional Ethics Committee of the Malabar Institute of Medical Sciences, Kozhikode, Kerala (IEC/2011/12). Blood samples were collected from fever patients according to Institute Ethics Committee guidelines and all participants gave written, informed consent. Total IgE was estimated (04827031190, Roche) by electrochemiluminescence immunoassay (Cobas e 411 analyzer, Roche).

### Western Blot Analysis

For protein isolation, fully emerged moths were ground and extracted with TEMP buffer (pH 8.0) containing 0.2 mM PMSF. The extracts were centrifuged at 12000 rpm for 15 min at 4°C and isolated proteins were kept at –20°C until use. Whole body extracts (50 *μ*g) were resolved on 12% SDS gel, stained with Coomassie Blue and photographed. Unstained gels were transferred (Trans-Blot Turbo, BioRad) on to PVDF membrane (Trans-Blot Turbo transfer pack, BioRad and Immobilon-P, Millipore), blocked with 3% BSA for 1 hour, blotted with patients’ sera (1:1 v/v in 1% BSA) overnight at 4°C, then blotted with monoclonal anti-human IgE-Fc (HRP) antibody 1:1000 dilution (ab99836, Abcam) at room temperature for 2 hours, followed by color development with diaminobenzidine (Sigma-Aldrich) and protein sizes were determined by standard markers (Promega and BioRad). Specific IgE inhibition was performed by pre-incubating patients’ sera (1:5 v/v in 3% BSA diluted in TBS-T) with 10–100 *μ*g of moth extract for 16 h at 4°C. The preincubated mixture was then added to the membrane containing separated moth proteins and blotting was performed. Immunoblot experiments were also performed by substituting fever patients’ sera with healthy control, IgE hypersensitive to other allergens (atopic dermatitis, dust and pollen) and fever control (IgE < 200 IU/mL) sera. Immunoblots with fever patients’ sera were performed on unrelated heterologous allergen extracts of cockroach and teak defoliator moth to check the specificity of the assay. For β-actin loading control, tiger moth total protein was separated on PAGE and transferred to PVDF membrane. After blocking with 3% BSA, the membrane was incubated with monoclonal anti-β-actin antibody (A2228, Sigma-Aldrich) overnight at 4°C. This was followed by blotting with secondary anti-mouse IgG AP-linked antibody (#7056, Cell Signaling) at room temperature for 3 hours. Finally the membrane was developed by BCIP/NBT (Sigma-Aldrich) method.

### Protein Sequencing

For sequencing, protein bands on the gel stained with Simply Blue safe stain (Invitrogen) were sliced and dehydrated with 2:1 mixture of acetonitrile (ACN): 50 mM ammonium bicarbonate (Sigma-Aldrich) and washed with 25 mM ammonium bicarbonate. Reduction and alkylation were done with 10 mM dithiothreitol (Sigma-Aldrich) and 100 mM idoacetamide (Sigma-Aldrich) respectively. Digestion was completed with trypsin (sequencing grade, Promega). Finally, extractions were carried out by increasing grade of extraction buffer having 50, 60 and 80% ACN containing 0.1% trifluroacetic acid (Sigma-Aldrich). After purification using ZIP-Tip, the solutions were loaded into the column (injection volume- 10.0 *μ*L, cap pump flow rate- 2.00 *μ*L/min, nano pump flow rate- 0.30 *μ*L/min) on HR-LCMS (1290 infinity UHPLC and Nano HPLC with 6550 iFunnel Q-TOF, Agilent). Sophisticated Analytical Instrument Facility operated by Indian Institute of Technology, Mumbai, India conducted the protein sequencing analyses.

### ELISA

Human TNF-α ELISA was estimated according to manufacturer’s protocol (KHC3011, Invitrogen Life Technologies). Briefly, added 50 *μ*L of incubation buffer to the pre-designated wells. Then added 100 *μ*L of standards, control and patients serum samples into the microtiter wells and incubated for two hours at room temperature. After washing four times, added 100 *μ*L of biotinylated anti-TNF-α (Biotin conjugate) solution into each well and incubated for one hour. After washing, 100 *μ*L of streptavidin-HRP working solution (4×) was added and incubated for thirty minutes. After washing, 100 *μ*L of stabilized chromogen was added to each well and incubated for thirty minutes in the dark. After incubation, samples were added with 100 *μ*L of stop solution and recorded the absorbance at 450 nm (iMark microplate reader, BioRad). Calculation was done using MasterPlex Reader Fit software (Hitachi).

### Animal Experiments

All animals were cared for in strict guidelines with Committee for the Purpose of Control and Supervision of Experiments on Animals (CPCSEA). The study was sanctioned by the Institute Animal Ethics Committee of the MIMS Research Foundation, Kozhikode, Kerala (MRF/IAEC/2012/#04). Male and female rats of Wistar and Sprague-Dawley strains (194.2 ± 27.4 gms, mean ± s.d, n = 48) were procured from Sri Chitra Tirunal Institute for Medical Sciences and Technology (SCTIMST), Thiruvananthapuram and College of Veterinary and Animal Sciences, Trissur, India. All animals were maintained at a controlled temperature of 26–28°C with a 12 h light:12 h dark cycle and fed with standard pellet diet and water ad libitum. Rats were challenged to live moths overnight in filtered cages provided with illuminated bright lights. Animals were monitored for the changes in the body including toenail or fingernail breaks, erythematous rash, bleeding eyes, ear or tail rash pedal edema and arthralgia. Rectal temperatures were recorded daily in the morning and evening using a digital thermometer. For platelet functioning study, 40 *μ*L of moth fluid (collected from prothoracic and abdominal points) was administered to each rat using a micropipette (5 *μ*L in alternate nostril at 2 min interval) in a supine position. Peripheral blood smears were prepared to verify the platelet properties prior and during the treatment and at the end of the recovery phase. Animals were sacrificed by terminal bleeding under ketamine (24 mg/kg, i/m) anesthesia. Blood samples were collected separately in K_2_-ETDA coated and serum collection tubes (BD Vacutainer). For serum collection, samples were centrifuged at room temperature and separated sera were stored at –20°C for further analysis. Complete blood count was performed using automated equipment (Coulter LH 780 analyzer, Beckman Coulter). Alanine aminotransferase and creatinine were estimated by photometric-P5P-kinetic and kinetic Jaffe reaction method respectively (Dimension EXL with LM, Siemens).

Rat IgE ELISA was performed according to manufacturer’s protocol (ab157736, Abcam). Briefly, added 100 *μ*L of each standard, control and toxin exposed rat serum samples into pre-designated wells and incubated the microtiter plates at room temperature for sixty minutes. After washing four times, added 100 *μ*L of 1× enzyme-antibody conjugate to each well and incubated for sixty minutes. After washing, added 100 *μ*L of tetramethylbenzidine (TMB) substrate solution and incubated in the dark for ten minutes. Reaction was stopped by adding 100 *μ*L of stop solution and determined the absorbance at 450 nm. Calculation was done using MasterPlex Reader Fit software.

### Tissue Processing and Staining

Small pieces of lungs, liver and kidneys were fixed in 10% buffered formalin and processed in paraffin (TP1020, Leica). Embedded tissues were sectioned (RM2245, Leica), stained with hematoxylin and eosin and examined pathological changes (double-blinded) under the microscope (ECLIPSE 80i & E200, Nikon). Toluidine blue stain was used for mast cell detection and Leishman-Giemsa was used for megakaryocyte detection in bone marrow and spleen respectively. The images were captured using Nikon DS Filc camera.

### Statistical Analysis

Chi-square analysis was performed to compare the symptoms among different age groups in the household survey conducted in fever patients. One-way ANOVA was performed to compare the blood sera parameters of healthy individuals with fever patients. Again, blood sera parameters of animals exposed to live tiger moths were compared with normal controls using one-way ANOVA and all the data are expressed as mean ± S.D. Cointegration and causality tests were carried out using EViews 8 and Gretl softwares to test the relationship of larval abundance with fever epidemics and environmental parameters.

## Results and Discussion

### Tiger Moth and Herbivory

**Fig 2:**
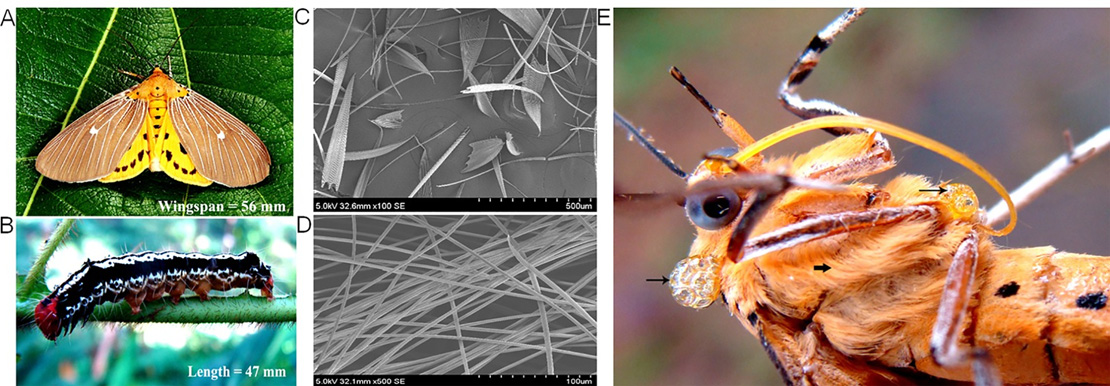
Tiger moth and toxic components. (A) *A*. *caricae* showing its characteristic white discal-spotted forewings and black-spotted yellow hind wings. (B) Final instar forage on pioneer host leaves is active at night but elusive during day light. Larva is blackish with red prothorax and a central black stripe in which on each segment two black spots arranged longitudinally (Ref. 18). The photographs were originally taken by P J Wills (Fig A & B). (C) Lamellar type represents majority of the scales on the head, thorax, wings and abdomen (magnification 100×) (D) Scales on the female ovipositor were mostly of piliform type (magnification 500×). (E) Defensive fluids released from specific bleeding points. Broad arrow show hairy tufts or “*flechettes*” (piliform type) and narrow ones denote the toxic secretions from teguments. The photograph was originally taken by Anjana Mohan (Fig E).

The dispersal of tiger moth is extensive from Asia to Pacific Islands [18] (Fig 1*A*) and we observed synchronous populations of moth in entire Kerala (Fig 1*B*) that are coinciding with massive fever epidemics (Fig 1*C*). The moth completes its life cycle in an average of 32.6 days with egg phase (2.7 ± 0.6 days; *n* = = 23 egg mass (S2*A* Fig), larval phase (S2*B* Fig) consist of five instars (12.9 ± 0.7; *n* = 120), pupae (9.5 ± 0.7; *n* = 157) and adult (7.5 ± 0.9; *n* = 136) (Fig 2*A*). New batches of eggs are laid in 3 days and thus we estimate a minimum generation time of 27 to 28 days. The folivorous caterpillars feed nocturnally on pioneer fig *Ficus hispida* (Fig 2*B*) and pupate on folded leaves (S2*C* Fig) or down in the earth. Adult ecloses during the dusk (18:00 to 19:30 hours) and the flight activity of both the genders are high during scotophase and fall to nil during photophase. Interestingly, female moths outnumber males in the populations (sex ratio 1:2.1, *n* = 643; χ^2^ = 77.2, *P* < 0.01) and mate multiple times. We observed short flights of moths during twilight followed by small aggregation with progression of nighttime and between 20:00 to 23:30 hours, tiger moths (6.4 ± 3.6 moths/aggregation, *n* = 92 observations, and range 2–13 moths) are attracted to artificial night lighting and light-coloured walls of human habitation.

Tiger moths render direct human contact in coherence with massive distribution and herbivory of pioneer figs in the region. *Ficus* represents an important component in terms of insect herbivory where the pioneer species suffer massive caterpillar attack while successional species receive less damage. This strategy is because pioneers tend more towards growth and are poorly defended whereas successional figs are well defended [19, 20]. Additionally, volatiles of the pioneers influence herbivory positively [21, 22] and caterpillars sequester these vital compounds (S3*A and S3B* Fig) to adults in blending chemicals that assist concomitant functions of the moth.

### Caterpillar Menace

Entry of tiger moth caterpillars, mostly final instars, into human habitation produced severe itching among children and elderly. Print media (*Malayala Manorama* June 12, 2012) reported large number of hospitalization when larval attack elicited wounds in children and worsened the condition due to severe itching in diabetic patients. We quantified the thick yellow fluid accrued in the caterpillar body estimated as ≈112.5 ± 8.7 *μ*L/caterpillar (*n* = 10) and found significant levels of imidazole (> 0.001 mg/*μ*L), diisooctyl phthalate and octan-1-ol (> 0.005 mg/*μ*L) and at this stage, caterpillar fluids could be passed on to the primary setae which serves as a defensive mechanism (18).

### Toxic Fluids and Scales of Tiger Moth

Aposematism or warning coloration, the survival tactic of moths, typically exhibits with combination of brilliant colors communicating a message about its bad taste or toxicity [22, 23]. Bestowed with dazzling colors, tiger moths flaunt their marked aposematism and if stimulated, dislodge scales, hairs or atomize body fluids to the ambient. The quantity of moth fluids released from prothoracic and abdominal points is estimated as ≈0.063 ± 0.07 gms/moth dry wt. (*n* = 8). We explored the moths’ arsenal and found that body fluids consisted of histamines (16.7 ± 13.3 mg/moth; *n* = 4), imidazole (1.1 ± 0.8 mg/moth; *n* = 4) (S4*A* Fig) and volatiles including octan-1-ol, 2-methyl-5-propan-2-ylcyclohexa-2,5-diene-1,4-dione and phthalates (S4*B* Fig). Moreover, we confirmed the existence of peptides in moth fluids (S5*A* and S5*B* Fig). The biogenic amines are vasoactive [24, 25], serve multiple functions with a tropism for receptors in bone, joints and cartilage resulting in chronic osteoarthritis, migratory polyarthritis and polychondritis [9]. Imidazole, a heterocyclic compound share 1,3-C3N2 ring system in histidine and histamine, is a diapause terminator [26] act as sturdy skin and eye irritants [27]. Further, octan-1-ol and phthalates could also be potential health hazards to human [28, 29].

Once tiger moths are attracted to artificial night lights in human habitation, they shed scales by vigorous wing fanning (S6*A* Fig), and/or discharge toxic fluids during physical or mechanical disturbances (S6*B* Fig). Scales are of two types, lamellar and piliform [30], are heavily distributed on the head, thorax, abdomen, wings and genitalia. Scales in various sizes (lamellar types, length 364.1 ± 222.2 *μ*m; maximum width 103.4 ± 41.0 *μ*m, *n* = 12 and piliform type, length 1125.7 ± 84.4 *μ*m; maximum width 4.3 ± 0.5 *μ*m, *n* = 12) are identified using scanning electron microscopy (Fig 2*C and 2D*). Furthermore, we observed that fluids released from specialized pores of prothoracic segments (Fig 2*E*) are spread over the hairy tufts and occasionally, these hairs are lifted by the long siphon of the proboscis and are dispersed into the air. We reckoned a maximum of twelve female moths attracted to human habitation in single occasion with full of detaching scales and hairs. Inhalation of the toxic components from a single moth is enough to cause the disease but cumulative effect of the toxins could generate severity of the disease, sometimes deadly. A 67-year-old female, one of the investigator’s relative, touched a live moth in a failed attempt during collection in the year 2009. She developed symptoms of the epidemic in just 24 hours and was bedridden with fever, intermittent chills and vomiting for the next two days. By day three, she had severe throat discomfort and generalized myalgia but subsided without any specific treatment. She was affected with the disease of similar symptoms in the year 2007 and was hospitalized for 4 days. Investigators (*n* = 3) were affected with the disease during the collection of live moths that attracted to artificial night lights. All of them developed the symptoms including fever, headache, back pain and generalized myalgia by the next day but the effects were transient.

### Heterogeneity of Tiger Moth Specific IgE in Human Sera

**Fig 3:**
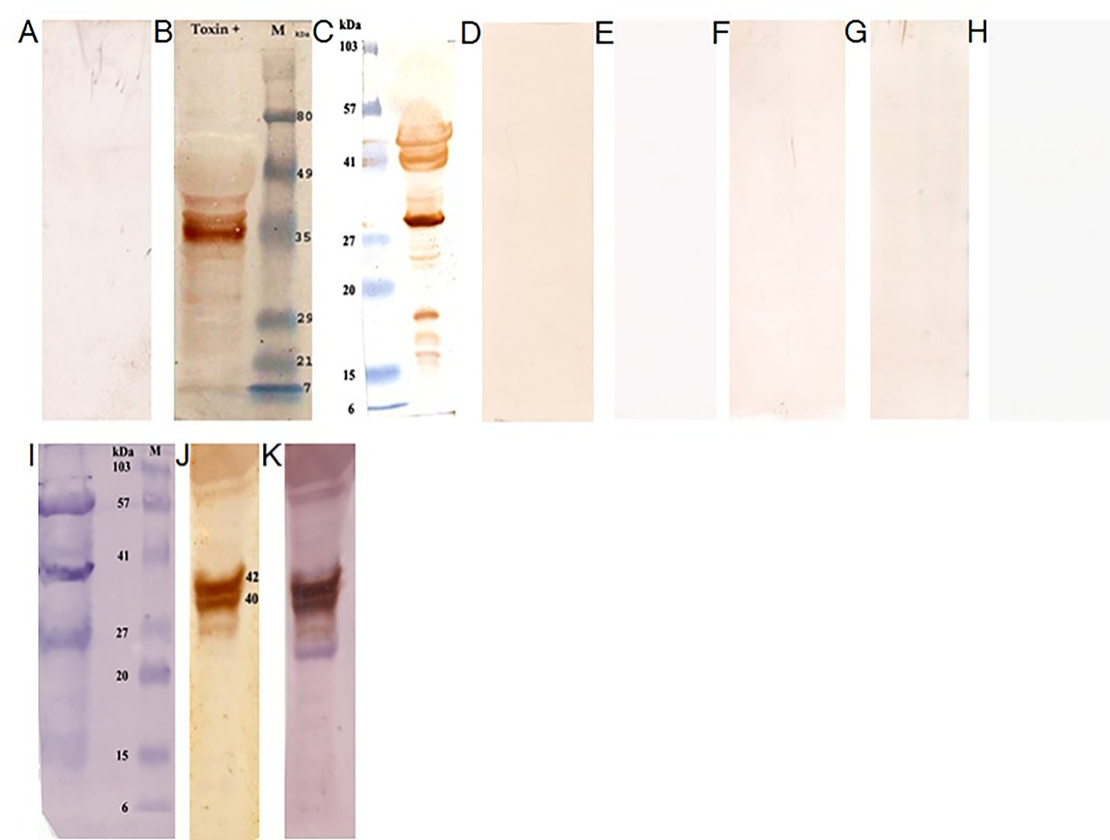
Tiger moth specific IgE in human sera. (A) Immunoblot shows no specific IgE reaction of healthy control serum against tiger moth total protein extract. (B) Specific IgE reaction of patient’s serum against tiger moth total protein extract. The patient was a 40 year old male diagnosed negative for chikungunya, HbsAg and leptospirosis but his total IgE level was > 2,500 IU/mL. (C) Another patient positive to tiger moth specific IgE. A 39 year old male presented with complaints of high grade fever, headache and myalgia. Basic bloods showed thrombocytopenia (≈52000) with deranged liver enzymes and marginally elevated creatinine levels. (D) No bands were detected on moth extract against IgE hypersensitive serum to other allergens (IgE level was > 2,500 IU/mL). (E) No specific IgE reaction of fever control serum (IgE level was < 200 IU/mL) against tiger moth total protein extract. (F) Serum pre-incubated with tiger moth protein show no specific IgE reaction but the same serum (not pre-incubated) showed tiger moth specific IgE response. (G) Serum from patient shows no specific IgE reaction against cockroach total protein but that showed a previously positive IgE response against tiger moth protein. (H) Serum from patient shows no specific IgE reaction against *Hyblea* moth total protein but that showed a previously positive IgE response against tiger moth total protein. (I) β-actin loading control. (J) Tiger moth specific 42 and 40 kDa IgE bands were prominent when blotted with fever patient serum. (K) The above membrane (J) was further blotted against monoclonal anti-β-actin antibody followed by blotting with secondary anti-mouse IgG AP-linked antibody. No further binding of β-actin was observed at 42 kDa region.

In a retrospective study, 83.5 percent of fever patients (*n* = 3,641) admitted to our hospital suspected with symptoms of infectious fevers through the years from 2009 to 2013 were serological negatives for chikungunya, dengue and leptospirosis. In contrast, the patients had elevated levels of total serum IgE (1,216.3 ± 737.5 IU/mL; *n* = 155 vs healthy control, 106.2 ± 65.0 IU/mL; *n* = 15, *P* < 0.05). Intriguingly, immunoblots with patients’ sera confirmed specific IgE-binding proteins that are congruent to tiger moths (78.6%, *n* = 61). No bands were found on tiger moth proteins against healthy control (n = 10), fever control (111.1 ± 70.7; *n* = 10), allergic control sera (886.0 ± 713.0, *n* = 17) and in specific IgE inhibition blots. Correspondingly, no bands were located on cockroach and *Hyblea* moth proteins against IgE elevated fever patients’ sera (*n* = 15 each) (Fig 3*A–3K*).

Five IgE-binding proteins were recognized as major allergens and the number of IgE-binding proteins detected on tiger moth extract varied among patients indicating heterogeneity in patients’ IgE response to moth allergens. Sequencing analyses highlight the diversity of IgE-binding proteins that include histone (11 kDa), triose-phosphate isomerase (27 kDa), 14-3-3ζ (30 kDa), arginine kinase (40 kDa) and actin (42 kDa). It is projected that these molecules play a key role in specific histamine release [31–35]. Similar IgE binding proteins have been identified in European pine processionary caterpillar *Thaumatopoea pityocampa* where, a 28 and 15 kDa IgE-binding protein toxin, thaumetopoein, serve as systemic histamine and kinin releaser [9].

### Long-Lasting Effect of the Disease

To understand the severity of the disease on different age groups, we conducted a survey among patients (*n* = 862) affected in the year 2007 with mean age of 36 years (range: 3.5–86) responded to the study variables (S1 Table). We observed that respondents with fever (*n* = 763) and joint pain (*n* = 824) did experience a greater risk of itch and/or rash (*n* = 591) with fever preceding the itch and/or rash rather than occurring concurrently. The most common report of the rash was blotchy red areas followed by hive-like bumps, locally named as “tomato fever”. Moreover, prolonged physical disabilities estimated to be the highest in elderly (≥ 60 years, *n* = 93), lower in adults (30 to 59 years, *n* = 254) and the lowest in children and young adults (< 30 years, *n* = 95) with no sex preference.

### Up Regulation of Tumor Necrosis Factor-α (TNF-α) in Crippling Arthralgia

In fever patients, the knee joint disease was distressingly persisting and even a slight touch or movement was debilitating. TNF-α concentrations are correlated with the severity of the joint disease, because of the more complex interaction of immune modulators at the synovial membrane [13, 36]. Based on these observations, we estimated levels of serum TNF-α concentrations, which showed significant difference between joint disease 287.7 ± 407.1 pg/mL (*n* = 26) and healthy control groups 17.2 ± 3.5 pg/mL (*n* = 10, *P* < 0.05). Joint damage or synovitis begins at the synovial membrane where there is an influx of mononuclear cells which produce inflammatory cytokines such as TNF-α and interleukins in the synovial fluid. These cytokines activate resident synovial cells to produce collagenase that mediate destruction of cartilage leading to the development, progression and severity of the disease [37].

### Tiger Moth Disease in Experimental Rats

**Fig 4:**
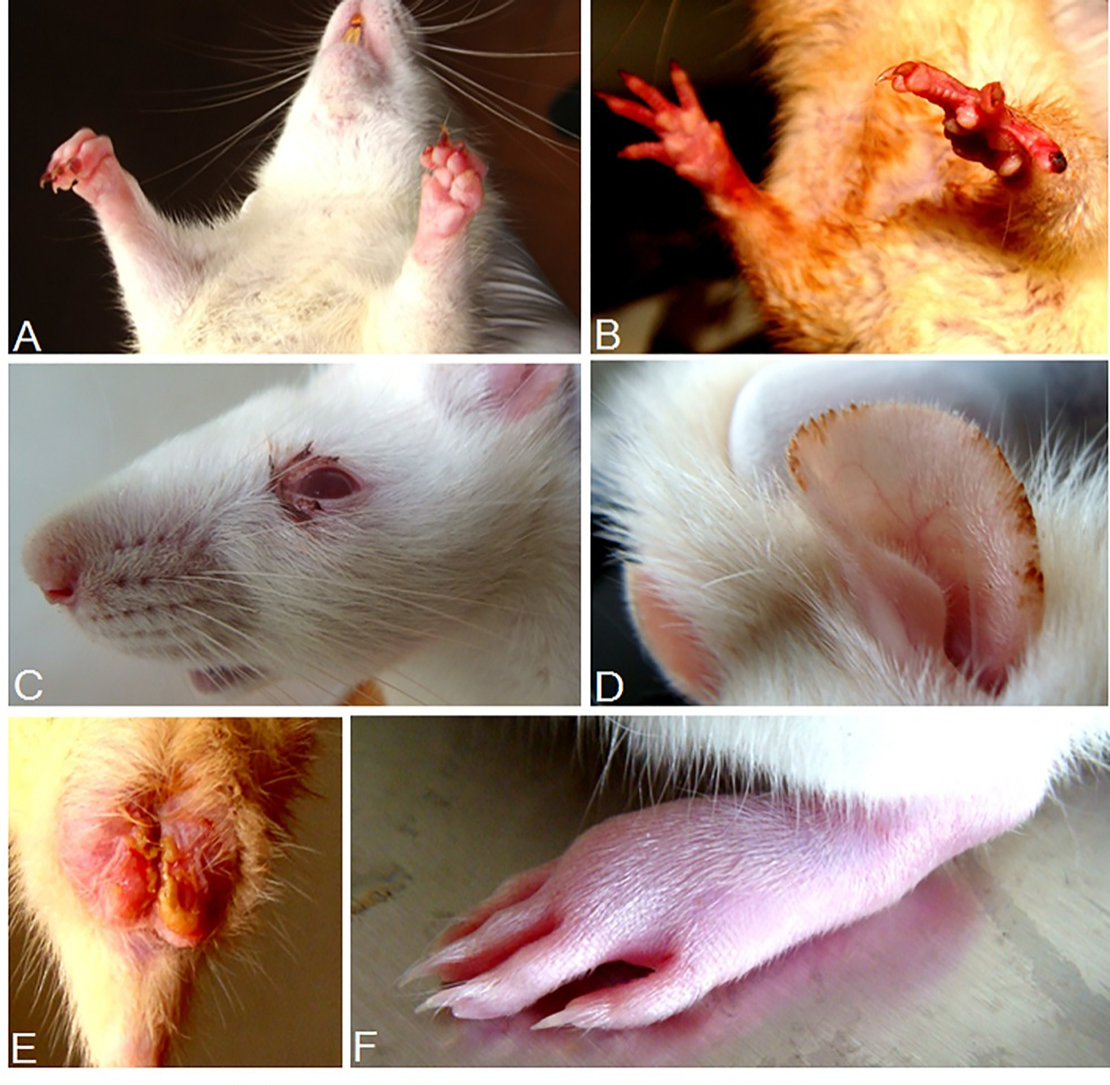
Tiger moth disease in experimental rats. Tiger moth toxin exposed rats produced symptoms that resembled fever patients affected during the epidemic. (A) Hemorrhagic syndrome leading to fingernail breaks on both hands, a sign leading to melanonychia. (B) Erythematous rash was prominent on both hands. (C) Bleeding eyes observed as an early sign of the disease. (D) Eruption of erythematous rash on ears. (E) Removal of soft skin on the testes. (F) Edema was confined to the legs and this presentation was very common among fever patients.

Experimental rats exposed to live moths (mean incubation time for 8 and 4 moths are 4.8 ± 2.0 and 9.5 ± 2.2 days respectively, *n* = 9) replicated the clinical signs of the epidemic that include severe itching (S1 Movie & S2 Dataset), fingernail breaks, erythematous rash, bleeding eyes, ear rash, removal of soft tissues, pedal edema (Fig 4*A*–4*F* & S7 Fig) and arthralgia (S2 Movie). Furthermore, rectal temperature (°F) elevated to 101.1 ± 0.4; *n* = 13 vs control rats (96.3 ± 1.9; *n* = 10, *P* < 0.05).

**Fig 5:**
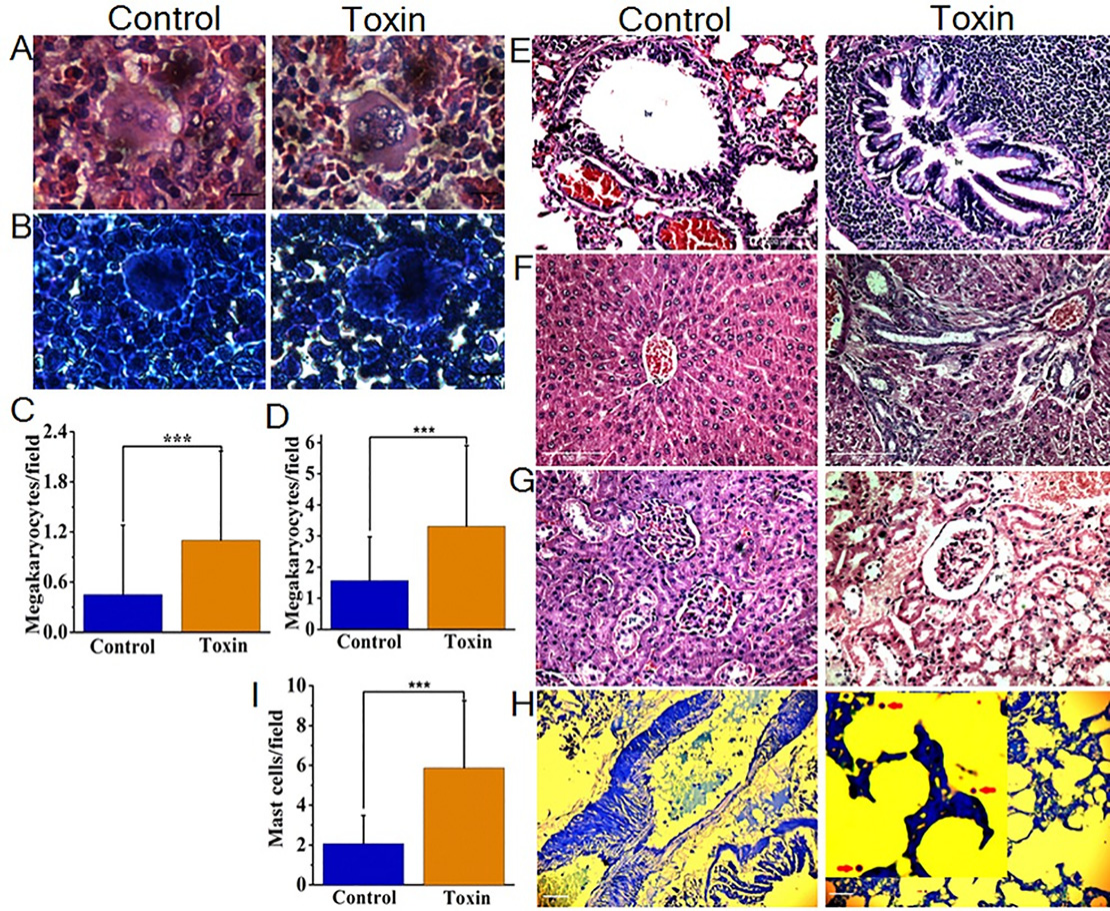
Toxic responses in experimental rats. (A-B) Regeneration of megakaryocytes in response to platelet reduction was evident with larger megakaryocytes with irregular multilobulated nucleus and more prominent nucleoli in spleen and bone marrow respectively. (C-D) Mean megakaryocyte volume represented by Leishman-Giemsa positive cells (per field@400×) in spleen (*n* = 60) and bone marrow (*n* = 120) respectively. (E) Pneumonitis caused due to extensive proliferation of lymphocytes in the peribronchial region (br, bronchiole). (F) Hepatic tissue reports centrilobular necrosis characterized by prominent ballooning, swollen granular cytoplasm with fading nuclei and nuclear infiltration (cv, central vein). (G) Mild acute renal tubular necrosis illustrated by destroyed cells in proximal tubules (pr). (H) Toluidine blue-positive mast cells (inset red arrow) scattered in lung tissue. (I) Mean mast cell recruitment (per field@400×) in lung tissue of control and toxin groups (*n* = 120). Asterisks indicate significant difference, *t*-test, *P <* 0.05. Fig A,B = magnification 1000× and scale bar = 8 *μ*m. Fig E-G = 200× and 100 *μ*m. Fig H = 400× and 20 *μ*m.

Finger or toenail breaks in experimental rats [38] was fixed as the criteria for defining the mean incubation time of rats subjected to intranasal administration of tiger moth toxin (40 *μ*L). It was estimated to be 8.1 ± 2.2 (*n* = 10) days. Complete blood count and peripheral blood smear evaluation revealed a significant decline in platelet count (7.7 ± 0.7 x10^5^/*μ*L, *n* = 10 vs control 10.7 ± 0.8 x10^5^/*μ*L, *n* = 10, *P* < 0.05) and the counts elevated during the recovery period (10.3 ± 0.8 x10^5^/*μ*L, *n* = 10, *P* < 0.05). Platelet morphology was normal with no platelet clumping indicates platelet activation and that also correlates with mean platelet volume. Regenerative responses to the decrease in the number of platelets and increased number of megakaryocytes in the splenic red pulp and bone marrow [39] were examined (Fig 5*A–5D*). In experimental rats, decline of platelets was significant but no deaths were recorded due to the toxicity of moth fluids. On an average, the platelet count in rats (10.7 x10^5^/*μ*L) is three times higher than that of humans and the decrease in platelets (3.0 x10^5^/*μ*L) might not have reached up to critical limit for the animal survival, but it can be fatal to human averaging 3.0 x10^5^/*μ*L in the circulation.

In addition, serum alanine aminotransferase elevated to 75.0 ± 13.7 IU/L; *n* = 10 vs control 55.0 ± 4.2 IU/L (*n* = 10, *P* < 0.05) and creatinine to 0.91 ± 0.1 mg/dl; n = 10 vs control 0.74 ± 0.1 mg/dl (*n* = 10, *P* < 0.05), indicating hepatic and renal injury. At necropsy (*n* = 5), lethal effect of the moth fluids leading to multi-organ toxicity was evident in histology findings (Fig 5*E–5G*). Fig 5*E* and S8*B* Fig demonstrate extensive lymphocytic proliferation in the peribronchial region and Fig 5*H* and 5*I* illustrate recruitment of mast cells in the bronchioles, both conditions have been reported to release pulmonary histamines [40, 41]. Most of the lepidopteran insects possess toxic chemicals such as histamines, and histamine releasing peptides that also facilitate immune-inflammatory responses [9*–*11, 34]. Interestingly, we found that progression of immune-inflammatory response mediated by the moth fluids has produced higher levels of rat specific IgE but precisely dependent on the quantity of the toxin exposed. Higher levels of IgE (224.0 ± 212.5 ng/mL, *n* = 15 vs control 12.3 ± 9.0 ng/mL, *n* = 10, *P* < 0.05) was quantified in the sera of rats exposed to moth toxins.

Lepidopteran scales consist of chitin, a polysaccharide biopolymer composed of *N*-acetyl-β-D-glucosamine, is a strong inducer of inflammatory reactions and potentiator of immune responses in mammals. Importantly, chitin compounds specifically binds to receptors on macrophages and activation process may lead to inflammatory reactions. Chitinase act on the chitin skeleton of the scales, and release fragmented chitin, proteins and other antigenic components. Proliferation of lymphocytes is initiated when these proteins are processed by antigen promoting cells, leading to allergic and other immune responses [42*–*45].

Late-phase reactions that follow early-phase reactions are thought to cause recurrent and chronic symptoms of allergic individuals. The release of histamines and other mast cell mediators such as interleukins, tumor necrosis factor-α (TNF-α and proteases contribute to initiate late-phase reaction by recruiting and activating inflammatory mediators, and ongoing dysregulation of such mediators cause organ dysfunction [46]. Experimental animals exposed to tiger moths are observed to have augmented recruitment of mast cells (Fig 5*H*) and severe perivascular lymphocytic proliferation in the lungs leading to bronchoconstriction (Fig 5*E* and S8*B* Fig) which could be the reason for respiratory distress and pneumonitis in patients observed during the epidemic. In addition, fever patients with joint disease had elevated levels of TNF-α, emphasize that severe arthralgia and edema with walking debility occur in the late-phase of the disease. Interestingly, a very similar and profound debility of joints with edema is observed in animal models (S1 Movie & S2 Movie) support the prevalence of chronic reactions in tiger moth lepidopterism. Precisely, these tissue damages have led to the development of chronic symptoms and severity of the disease, suggest that mechanisms other than those mediated by IgE are also at play.

### Tiger Moth Waves Cointegrate with Epidemics

We observed that the incidence of epidemics may occur either by aggregation of local endemic moth population or by immigration from a place under larval attacks. Thus, metapopulation theory better explains the population dynamics of tiger moth explicitly [47]. Interestingly, the moths’ haplotypes represent against vicariance that located in Pacific region at a distance of 5,340 km covering Taiwan→New Guinea→Australia suggesting long-distance dispersal of the species [48]. To look for the magnitude of moth population, we used field estimates of caterpillars on pioneer figs and observed successive generations of moths in the area, pointing to the fact that imidazole play a pivotal role in the termination of diapause entry [26]. Here erratic outbreaks occur from January to April, but aggregate as mild wave in May, followed by huge waves from June to August, which then decline in September through December. Therefore, the outbreak of caterpillars appears to be eruptive type exemplifying a positive density-dependent feedback mechanism [49, 50].

Repeatedly, epidemics start three to six days after adult moths’ eclosion and turn to huge epidemics when populations overlap. To address the direct relation between moth emergence and fever epidemic, we conducted a time series model of larval population against disease incidence (Fig 1C) using the cointegration analysis. We further integrated meteorological conditions with moth abundance and found the existence of cointegration relation between relative humidity, temperature, rainfall and larval abundance (Table 1) using Augmented Dickey-Fuller test [51, 52]. At 0.05 level, the cointegration rank test suggests the existence of at most four cointegrating vectors in the system (Table 2) by methods of Johansen and Juselius [53]. Further, Granger causality test [54] indicate that there is unidirectional causality that drives larval abundance to fever epidemics (F-Statistic = 15.25, *P* = 0.00001) and the larval abundance is Granger caused by atmospheric temperature (F-Statistic = 6.54, *P* = 0.003).

**Table 1:** Time series properties of variables and the test suggest that all the series are stationary.

Variable	Augmented Dickey-Fuller test statistic	P value*
Temperature	(-6.02)	0.0000
Rainfall	(-3.50)	0.0120
Relative humidity	(-5.39)	0.0001
Larvae	(-5.14)	0.0001
Fever	(-4.65)	0.0005

Values that exceed the 0.05 threshold (-2.9) are in parenthesis. The 1% critical value is -3.6

Stationary of order I(1)

*MacKinnon (1996) one-sided p-values.

**Table 2:** Unrestricted Cointegration Rank Test (Trace) by methods of Johansen and Juselius.

Hypothesized Number of Cointegrating Equations(CE)	Eigenvalue	Trace Statistic	0.05 Critical Value	Prob.**
None *	0.660	121.22	69.82	0.0000
At most 1 *	0.440	71.54	47.86	0.0001
At most 2 *	0.360	44.87	29.80	0.0005
At most 3 *	0.284	24.32	15.49	0.0018
At most 4 *	0.177	8.96	3.84	0.0028^a^

Sample (adjusted): 2009–2012.

Included observations: 46 after adjusting endpoints.

* denotes rejection of the hypothesis at the 0.05 level.

** MacKinnon-Haug-Michelis (1999) p-values.

Trace test indicates 5 cointegrating eqn(s) at the 0.05 level.

^a^The p-value associated five sets of CE suggest that all the four variables are co-integrated with the variable fever.

Relative humidity governs the development and survival of insects since the body water content needs to be kept at equilibrium. This is perhaps important for the early instars (S2*B* Fig) which survive better and develop faster when aggregated, possibly to avoid desiccation [55]. Being ectotherms, insects face greatest extinction risks in the absence of either migration or adaptation (S9 Fig) [56*–*58]. We found that tiger moths have high survival and reproductive strategies, balancing the populations to 30.1 ± 5.7 eggs/moth (*n* = 21) at minimum relative humidity (< 82%) and high temperature (> 32.0°C). Besides, it is reasonable to consider maximum humidity (> 82%) and optimum temperature (< 32.0°C) as abiotic factors influencing the fitness that increased the fecundity (171.6 ± 21.8 eggs/moth, *n* = 19), implying mating success of the moth. In this circumstance, deluge of caterpillars emerge from egg masses that infest the host plants and eventually the disparity in larval growth rate increases between first instars and prepupae stages that plausibly is the reason for overlapping populations of the moth.

## Conclusion

During the recent fever outbreaks in Kerala, a large group of patients had clinical symptoms similar to chikungunya and dengue. Interestingly, a significant number of the cases were negative to these infectious fevers and clinicians treated the disease symptomatically. In any of these fever outbreaks, the association between adult tiger moths and the epidemics were not taken in to account and most of these cases were classified as suspected infectious fevers. In clinics, delay in treatment due to a series of investigative procedures had complicated the conditions including platelet drops, respiratory disorders, meconium aspiration syndrome, hepatic and renal failure leading to a number of deaths. In this context, our findings would provide new insights in the development of applied clinical treatment for the fever patients and avert large number of deaths owing to symptomatic treatment that are being currently followed in the State. Improved surveillance networks are required to track the movement of the tiger moths associated to different seasons in other regions where also similar epidemics have been reported. Real-time surveillance and warning system during the build-up phase of caterpillars and pruning host plants by properly trained personnel could manage heavy invasion of the tiger moth populations.

## Supporting Information

S1 Dataset GPS coordinates. Geographical coordinates of the study area that represents all the fourteen districts of Kerala where host plants infested with tiger moth caterpillars were recorded from July to September in the year 2009. We also confirm that the GPS coordinates contained with our supporting information files will not lead to the identification of any specific individuals.
(XLS)

S2 Dataset Frequency and duration of itching in rats. Rats exposed to live moths had severe itching all over the body. Frequency and duration of itching was significant in experimental rats exposed to live tiger moths compared to unexposed rats.
(XLSX)

S1 Fig Epidemics in Kerala. Average monthly variation of fever occurrence from the year 2006 to 2014. Till September 01, 2015, 10141 suspected dengue (including 33 deaths), 1420 suspected leptospirosis (including 54 deaths) and 116 suspected chikungunya cases were reported in the current year. Source: Directorate of Health Services, Public Health, Kerala. Available at http://dhs.kerala.gov.in/index.php/publichealth.
(TIF)

S2 Fig Egg mound, early instars and cocoon. (A) Eggs are laid in domed clusters on the ventral side of host leaves. The hairy *flechettes* and/or anal tufts are used to protect the eggs from predators. (B) Early instars aggregate on the leaves to avoid dehydration. These instars feed on younger leaves and later move on to older leaves. (C) Pupa in a curled *Ficus* leaf (viability 96.4%, *n* = 852). At temperature below 32°C, the larvae fold the leaf tips with its silk to make the cocoons whereas pupates in loose soil during high temperatures. The photographs were originally taken by P J Wills, corresponding author.
(TIF)

S3 Fig Chemical profiling of caterpillar hemolymph. (A) GC-MS analysis demonstrates the presence of octan-1-ol (Rt 5.76 min) and diisooctyl phthalate (Rt 11.08 min) in late fifth instars. (B) Diisooctyl phthalate identified in the hemolymph of fourth instars (Rt 11.08 min) but octan-1-ol was absent. The GC column DB-5 was used for the analysis. We quantified the thick yellow fluid accrued in the caterpillar body estimated as ≈112.5 ± 8.7 *μ*L/caterpillar (*n* = 10) and found significant levels of imidazole (> 0.001 mg/*μ*L), diisooctyl phthalate and octan-1-ol (> 0.005 mg/*μ*L).
(TIF)

S4 Fig Chemical profile of secretions obtained from tiger moth. (A) HPLC purified fraction identified as (*1*) histamine, (*2*) 4-methyl histamine, and (*3*) imidazole in tiger moth secretions by GC-MS equipped with a fused silica column. (B) GC-MS analysis identified major volatile compound as 2-methyl-5-propan-2-ylcyclohexa-2,5-diene-1,4-dione (32.8 min) in just emerged moths. The presence of octan-1-ol is detected at 19.9 min. The total content of the octan-1-ol was quantified (0.01 to 1.3%) using standard octanol. 1,4-Benzenedicarboxylic acid, dimethyl ester is identified at 43.69 min. The GC column DB-5ms was used for the analysis. Both females and males release body fluids into the ambient, and females produce more quantity of fluids than males due to the bigger size but qualitatively both male and female secretions are same.
(TIF)

S5 Fig Peptides in tiger moth fluids. (A) MALDI-TOF-MS analysis demonstrates the presence of peptides (0.5 to 1.4 kDa) in moth secretion. (B**)** Presence of different peptides including a 4.2 kDa peptide in moth excretion.
(DOCX)

S6 Fig Tiger moth scales and toxic fluids. (A) Powdery scales are easily detachable with aging. (B) Cream colored thick fluid is discharged from abdominal and yellow-coloured liquid is discharged from prothoracic points. The photographs were originally taken by P J Wills, corresponding author. We reckoned a maximum of twelve female moths attracted to human habitation in a single occasion with full of detaching scales and hairs. Inhalation of the toxic components from a single moth is enough to cause the disease but cumulative effect of the toxins could generate severity of the disease, sometimes deadly.
(TIF)

S7 Fig Quantification of symptoms. The animals were exposed to 8 and 4 moths in two groups and each group consisted of 9 animals each (100% = 18 animals). A represent percentage of finger nail/toenail breaks, B-erythematous rash, C- bleeding eyes, D-ear rash, E-peeling of testicular skin, F-edema.
(TIF)

S8 Fig Lymphocytic proliferation in the lungs. (A) Normal rat lung, (B) Inflamed lung represent extensive perivascular lymphocytic proliferation leading to bronchoconstriction. Low-power view (magnification 40×). Scale bar = = 400 *μ*m.
(TIF)

S9 Fig Tiger moth infested host plant. Tiger moth caterpillars select cooler microhabitats during warmest hours to avoid maximum operative temperatures. At high temperatures, female tiger moths prefer to lay eggs on host plants that are covered by twining climbers provide microclimate for the growing instars which function as a perfect shelter from soaring temperatures. With the arrival of monsoon, the caterpillars forage on all level of pioneer habits due to the changes in atmospheric temperature, leading to large scale outbreaks of tiger moths. The photograph was taken by P J Wills.
(TIF)

S1 Movie Severe itching. Rat exposed to live moths developed severe itching all over the body. Also observed severe edema on the right leg.
(MP4)

S2 Movie Severe arthralgia. Rat exposed to live moths developed severe edema and crippling arthralgia. Debility to walk sustained for 3 to 5 days and recovered by self. Similar presentation was widespread in fever patients during the epidemic.
(MP4)

S1 Table Comparison of symptoms (%) among different age groups (yr) during 2007 fever outbreak in Kerala, India. (DOCX)

S2 Table Diagnostic tests/platform used to screen different infectious fever diseases at MIMS. (DOCX)

## References

[1] World Health Organization.Chikungunya in India. Available: *http://www.who.int/csr/don/2006_10_17/en/*. Accessed 4 June 2014.

[2] Enserink M Infectious diseases. Chikungunya: no longer a third world disease. Science 2007; 318: 1860–1861. 18096785 10.1126/science.318.5858.1860

[3] Directorate of Health Services, Public Health, Kerala. Available: *http://dhs.kerala.gov.in/index.php/publichealth*. Accessed 1 September 2015.

[4] Kannan M, Rajendran R, Sunish IP, Balasubramaniam R, Arunachalam N, Paramsivan R, et al A study on chikungunya outbreak during 2007 in Kerala, south India. Indian J Med Res 2009; 129: 311–315. 19491425

[5] Sudeep AB, Parashar D Chikungunya: an overview. J Biosci 2008; 33: 443–449. 19208970 10.1007/s12038-008-0063-2

[6] Nair PMC Chikungunya in Neonates. Indian Pediatr 2008; 45: 605. 18695288

[7] Varshney V Chikungunya chase. Down to Earth 2008; 17: 38–40.

[8] Hossler EW Caterpillars and moths. Dermatol Ther 2009; 22: 353–366. 10.1111/j.1529-8019.2009.01247.x 19580579

[9] Diaz JH The evolving global epidemiology, syndromic classification, management, and prevention of caterpillar envenoming. Am J Trop Med Hyg 2005; 72: 347–357. 15772333

[10] Carrijo-Carvalho LC, Chudzinski-Tavassi AM The venom of the *Lonomia* caterpillar: An overview. Toxicon 2007; 49: 741–757. 17320134 10.1016/j.toxicon.2006.11.033

[11] Jourdain F, Girod R, Vassal JM, Chandre F, Lagneau C, Fouque C, et al The moth *Hylesia metabus* and French Guiana lepidopterism: centenary of a public health concern. Parasite 2012; 19: 117–128. 22550622 10.1051/parasite/2012192117 PMC3671431

[12] Villas-Boas IM, Gonçalves-de-Andrade RM, Pidde-Queiroz G, Assaf SL, Portaro FC, Sant'Anna OA, et al *Premolis semirufa* (Walker, 1856) Envenomation, Disease Affecting Rubber Tappers of the Amazon: Searching for Caterpillar-Bristles Toxic Components. PLoS Negl Trop Dis 2012; 6: e1531 10.1371/journal.pntd.0001531 22389740 PMC3289609

[13] Villas-Boas IM, Gonçalves-de-Andrade RM, Squaiella-Baptistão CC, Sant'Anna OA, Tambourgi DV Characterization of phenotypes of immune cells and cytokines associated with chronic exposure to *Premolis semirufa* caterpillar bristles extract. PLoS One 2013; 8: e71938 10.1371/journal.pone.0071938 24023721 PMC3762804

[14] Malaque CM, Andrade L, Madalosso G, Tomy S, Tavares FL, Seguro AC, et al Short report: A case of hemolysis resulting from contact with a *Lonomia* caterpillar in southern Brazil. Am J Trop Med Hyg 2006; 74: 807–809. 16687684

[15] Huang DZ Dendrolimiasis: an analysis of 58 cases. J Trop Med Hyg 1991; 94: 79–87. 2023292

[16] Webb BA, Barney WE, Dahlman DL, DeBorde SN, Weer C, Williams NM, et al, Eastern tent caterpillars (*Malacosoma americanum*) cause mare reproductive loss syndrome. J Insect Physiol 2004; 50: 185–193. 15019520 10.1016/j.jinsphys.2003.11.008

[17] Cawdell-Smith AJ Equine amnionitis and fetal loss: mare abortion following experimental exposure to Processionary caterpillars (*Ochrogaster lunifer*). Equine Vet J 2012; 44: 282–288. 10.1111/j.2042-3306.2011.00424.x 21815917

[18] Holloway JD. The Moths of Borneo: Family Arctiidae, Subfamilies Synthominae, Euchromiinae, Arctiinae; Noctuidae misplaced in Arctiidae, Camptoloma, Aganainae, 1988. Southdene Sdn Bhd, Kuala Lumpur, Malaysia. pp 85–89.

[19] Basset Y, Novotny V, Weiblen G *Ficus*: a resource for arthropods in the tropics, with particular reference to New Guinea: Forests and Insects, 1997 Chapman & Hill, London pp 341–361.

[20] Xiang H, Chen J Interspecific variation of plant traits associated with resistance to herbivory among four species of *Ficus* (Moraceae). Ann Bot 2004; 94: 377–384. 15277244 10.1093/aob/mch153 PMC4242178

[21] Song Q, Yang D, Zhang G, Yang C Volatiles from *Ficus hispida* and their attractiveness to fig wasps. J Chem Ecol 2001; 27: 1929–1942. 11710602 10.1023/a:1012226400586

[22] Nishida R Sequestration of defensive substances from plants by Lepidoptera. Annu Rev Entomol 2002; 47: 57–92. 11729069 10.1146/annurev.ento.47.091201.145121

[23] Weller SJ, Jacobsen NL, Conner WE The evolution of chemical defenses and mating systems in tiger moths (Lepidoptera: Arctiidae). Biol J Linn Soc 1999; 68: 557–578.

[24] Binstadt BA, Patel PR, Alencar H, Nigrovic PA, Lee DM, Mahmood U, et al Particularities of the vasculature can promote the organ specificity of autoimmune attack. Nat Immunol 2006; 7: 284–292. 16444258 10.1038/ni1306

[25] Mokry J, Mokra D, Nosalova G Effects of meconium on airway reactivity to histamine and acetylcholine in vitro. J Physiol Pharmacol 2007; 58: 409–417. 18204153

[26] Li Y, Xia RX, Wang H, Li XS, Liu YQ, Wei ZJ, et al Construction of a full-length cDNA Library from Chinese oak silkworm pupa and identification of a KK-42-binding protein gene in relation to pupa-diapause termination. Int J Biol Sci 2009; 5: 451–457. 19564928 10.7150/ijbs.5.451 PMC2702828

[27] UNEP Publications. Imidazole, OECD SIDS, Available: *http://www.inchem.org/documents/sids/sids/288324.pdf*. Accessed 3 March 2009.

[28] US National Library of Medicine 1-Octanol. Human Health Effects. TOXNET, Toxicology Data Network. Available: http://toxnet.nlm.nih.gov/cgi-bin/sis/search2/r?dbs+hsdb:@term+@DOCNO+700. Accessed 9 September 2014.

[29] Pan TL, Wang PW, Aljuffali IA, Hung YY, Lin CF, Fang JY Dermal toxicity elicited by phthalates: evaluation of skin absorption, immunohistology, and functional proteomics. Food Chem Toxicol 2014; 65: 105–114. 10.1016/j.fct.2013.12.033 24384410

[30] Scoble MJ The Lepidoptera: Form, Function, and Diversity, 1992 Oxford University Press/Natural History Museum pp 63–66.

[31] Tasaka K, Mio M, Akagi M, Saito T Histamine release induced by histone and related morphological changes in mast cells. Agents Actions 1990; 30: 114–117. 1695426 10.1007/BF01969013

[32] Moon J-A, Kim H-J, Lee K Interaction between IgE-dependant histamine releasing factor and triosephosphate isomerase in HeLa cells. Kor J Microbiol and Biotechnol 2005; 33: 255–259.

[33] Fujii T, Ueeda T Stimulation of 14-3-3 protein and its isoform on histamine secretion from permeabilized rat peritoneal mast cells. Biol Pharm Bull 2002; 25: 1524–1527. 12499633 10.1248/bpb.25.1524

[34] Binder M, Mahler V, Hayek B, Sperr WR, Schöller M, Prozell S, et al Molecular and immunological characterization of arginine kinase from the Indian meal moth, *Plodia interpunctella*, a novel cross-reactive invertebrate pan-allergen. J Immunol 2001; 167: 5470–5477. 11673567 10.4049/jimmunol.167.9.5470

[35] Tasaka K, Akagi M, Miyoshi K Distribution of actin filaments in rat mast cells and its role in histamine release. Agents Actions 1986; 18: 49–52. 2425590 10.1007/BF01987980

[36] Hess A, Axmann R, Rech J, Finzel S, Heindl C, Kreitz S A, et al Blockade of TNF-α rapidly inhibits pain responses in the central nervous system. Proc Natl Acad Sci U S A 2011; 108: 3731–3736. 10.1073/pnas.1011774108 21245297 PMC3048151

[37] Joosten LA, Netea MG, Kim SH, Yoon DY, Oppers-Walgreen B, Radstake TR, et al IL-32, a proinflammatory cytokine in rheumatoid arthritis. Proc Natl Acad Sci U S A 2006; 103: 3298–3303. 16492735 10.1073/pnas.0511233103 PMC1413916

[38] Rudmann DG, Page TJ, Vahle JL, Chouinard L, Haile S, Poitout F, et al Rat-specific decreases in platelet count caused by a humanized monoclonal antibody against sclerostin. Toxicol Sci 2012; 125: 586–594. 10.1093/toxsci/kfr318 22106037

[39] Fortoul TI, Piñón-Zarate G, Diaz-Bech ME, González-Villalva A, Mussali-Galante P, Rodriguez-Lara V, et al Spleen and bone marrow megakaryocytes as targets for inhaled vanadium. Histol Histopathol 2008; 23: 1321–1326. 18785114 10.14670/HH-23.1321

[40] Blaszczyk E B Mast cells as a source and target for histamine In Biomedical Aspects of Histamine: Current Perspectives, 2010 Springer Science Business Media B.V. NY. pp 247–284.

[41] Khan MM Effects of histamine on lymphocytes In Biomedical Aspects of Histamine: Current Perspectives, 2010 Springer Science Business Media B.V. NY. pp 151–174.

[42] Battisti A, Holm G, Fagrell B, Larsson S. Urticating hairs in arthropods: their nature and medical significance. Annu Rev Entomol 2011; 56: 203–220. 10.1146/annurev-ento-120709-144844 20809805

[43] Lee CG, Da Silva CA, Lee JY, Hartl D, Elias JA Chitin regulation of immune responses: an old molecule with new roles. Curr Opin Immunol 2008; 20: 684–689. 10.1016/j.coi.2008.10.002 18938241 PMC2605627

[44] Da Silva CA, Chalouni C, Williams A, Hartl D, Lee CG, Elias JA, et al Chitin is a size dependent regulator of macrophage TNF and IL-10 production. J Immunol 2009; 6: 3573–3582. 10.4049/jimmunol.0802113 19265136

[45] Santos-Magadán S, González de Olano D, Bartolomé-Zavala B, Trujillo-Trujillo M, Meléndez-Baltanás A, González-Mancebo E Adverse reactions to the processionary caterpillar: irritant or allergic mechanism? Contact Dermatitis 2009; 60: 109–110. 10.1111/j.1600-0536.2008.01464.x 19207385

[46] Bischoff SC Role of mast cells in allergic and non-allergic immune responses: comparison of human and murine data. Nat Rev Immunol 2007; 7: 93–104. 17259966 10.1038/nri2018

[47] Hanski I Metapopulation dynamics. Nature 1998; 396: 41–49.

[48] Craft KJ, Pauls SU, Darrow K, Miller SE, Hebert PD, Helgen LE, et al Population genetics of ecological communities with DNA barcodes: An example from New Guinea Lepidoptera. Proc Natl Acad Sci U S A 2010; 107: 5041–5046. 10.1073/pnas.0913084107 20202924 PMC2841870

[49] Nair KSS. Tropical forest insect pests: ecology, impact and management, 2007 Cambridge University Press, NY pp 119–133.

[50] Raffa KF, Powell EN, Townsend PA Temperature-driven range expansion of an irruptive insect heightened by weakly coevolved plant defenses. Proc Natl Acad Sci U S A 2013; 110: 2193–2198. 10.1073/pnas.1216666110 23277541 PMC3568305

[51] Dickey DA, Fuller WA Distribution of estimators for autoregressive time series with a unit root. J Am Stat Assoc 1979; 74: 427–431.

[52] Kaufmann RF, Stern DI Cointegration analysis of hemispheric temperature relations. J Geophys Res 2002; 107: 1–10.

[53] Johansen S, Juselius K Maximum likelihood estimation and inference on cointegration with application to the demand for money. Oxford Bull Econ Stat 1990; 52: 169–210.

[54] Granger CWJ Investigating causal relations by econometric models and cross-spectral methods Econometrica 1969; 37: 424–438.

[55] Willmer PG. Microclimate and the environmental physiology of insects Advances in Insect Physiology, 1982 Academic Press, London pp 1–57.

[56] Nelson WA, Bjørnstad ON, Yamanaka T Recurrent insect outbreaks caused by temperature-driven changes in system stability. Science 2013; 341:796–799. 10.1126/science.1238477 23907532

[57] Deutsch CA, Tewksbury JJ, Huey RB, Sheldon KS, Ghalambor CK, Haak DC, et al Impacts of climate warming on terrestrial ectotherms across latitude. Proc Natl Acad Sci U S A 2008; 105: 6668–6672. 10.1073/pnas.0709472105 18458348 PMC2373333

[58] Sunday JM, Bates AE, Kearney MR, Colwell RK, Dulvy NK, Longino JT, et al Thermal-safety margins and the necessity of thermoregulatory behavior across latitude and elevation. Proc Natl Acad Sci U S A 2014; 111: 5610–5615. 10.1073/pnas.1316145111 24616528 PMC3992687

