# Supplementary figures and images for: Population Explosions of Tiger Moth Lead to Lepidopterism Mimicking Infectious Fever Outbreaks
Source: PLoS One. 2016 Apr 13;11(4):e0152787. doi: 10.1371/journal.pone.0152787 (PMC4830441; doi:10.1371/journal.pone.0152787)

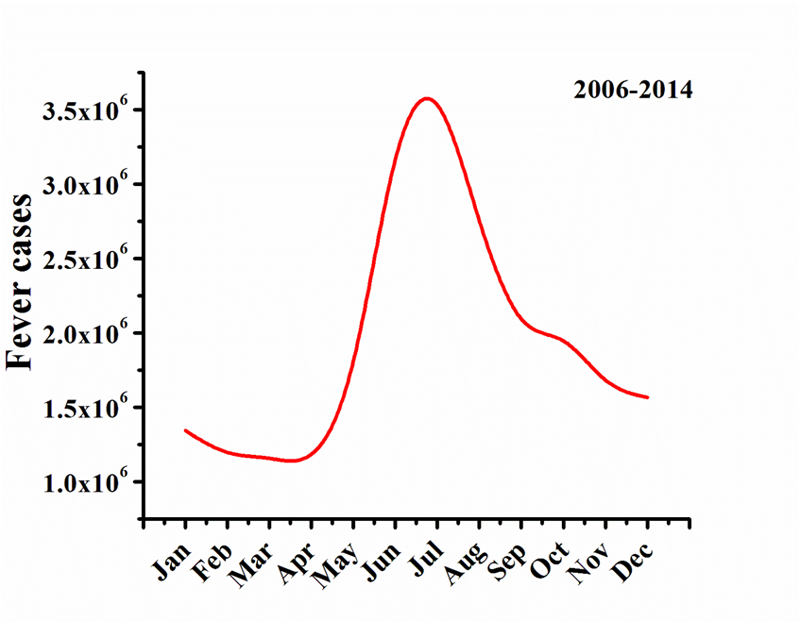

Supplement: S1 Fig — Average monthly variation of fever occurrence from the year 2006 to 2014. Till September 01, 2015, 10141 suspected dengue (including 33 deaths), 1420 suspected leptospirosis (including 54 deaths) and 116 suspected chikungunya cases were reported in the current year. Source: Directorate of Health Services, Public Health, Kerala. Available at http://dhs.kerala.gov.in/index.php/publichealth. (TIF) [file pone.0152787.s003.tif]

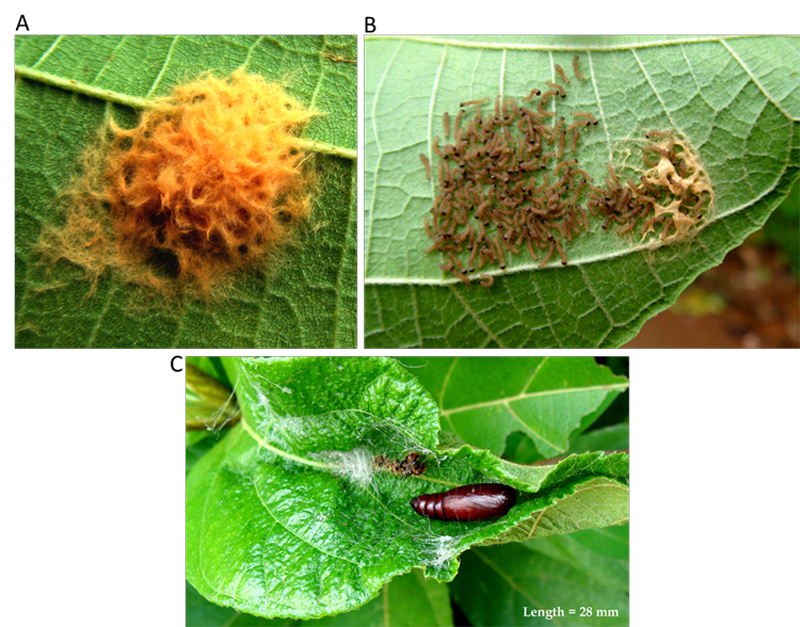

Supplement: S2 Fig — (A) Eggs are laid in domed clusters on the ventral side of host leaves. The hairy flechettes and/or anal tufts are used to protect the eggs from predators. (B) Early instars aggregate on the leaves to avoid dehydration. These instars feed on younger leaves and later move on to older leaves. (C) Pupa in a curled Ficus leaf (viability 96.4%, n = 852). At temperature below 32°C, the larvae fold the leaf tips with its silk to make the cocoons whereas pupates in loose soil during high temperatures. The photographs were originally taken by P J Wills, corresponding author. (TIF) [file pone.0152787.s004.tif]

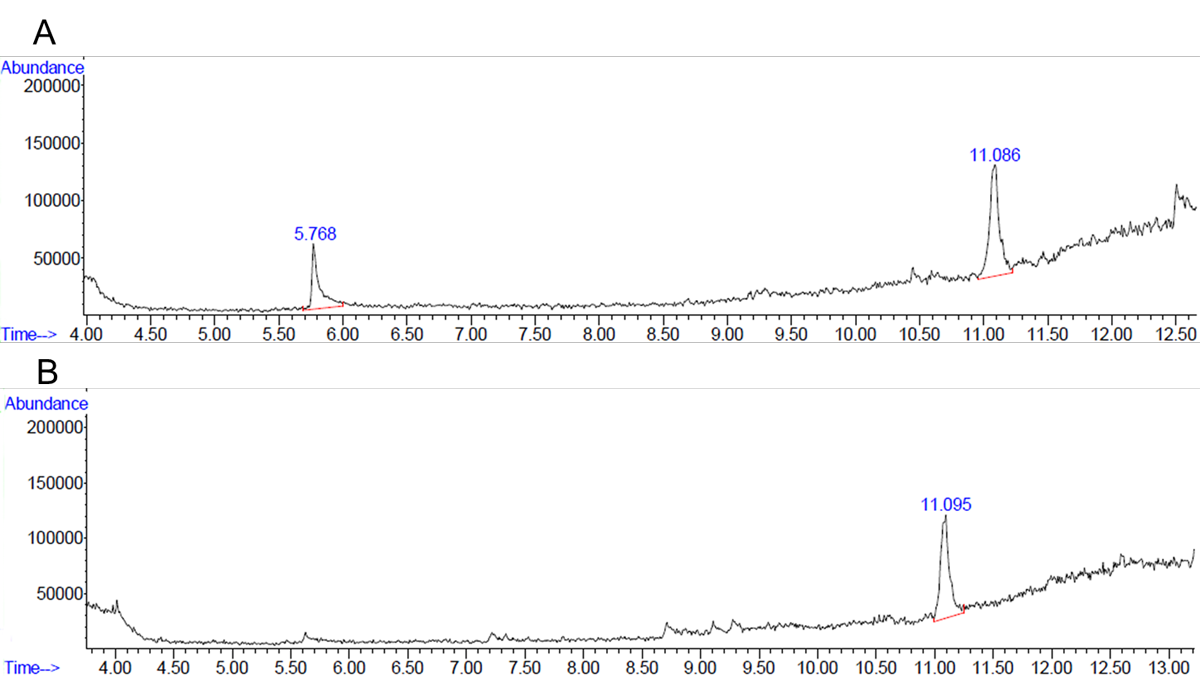

Supplement: S3 Fig — (A) GC-MS analysis demonstrates the presence of octan-1-ol (Rt 5.76 min) and diisooctyl phthalate (Rt 11.08 min) in late fifth instars. (B) Diisooctyl phthalate identified in the hemolymph of fourth instars (Rt 11.08 min) but octan-1-ol was absent. The GC column DB-5 was used for the analysis. We quantified the thick yellow fluid accrued in the caterpillar body estimated as ≈112.5 ± 8.7 μL/caterpillar (n = 10) and found significant levels of imidazole (> 0.001 mg/μL), diisooctyl phthalate and octan-1-ol (> 0.005 mg/μL). (TIF) [file pone.0152787.s005.tif]

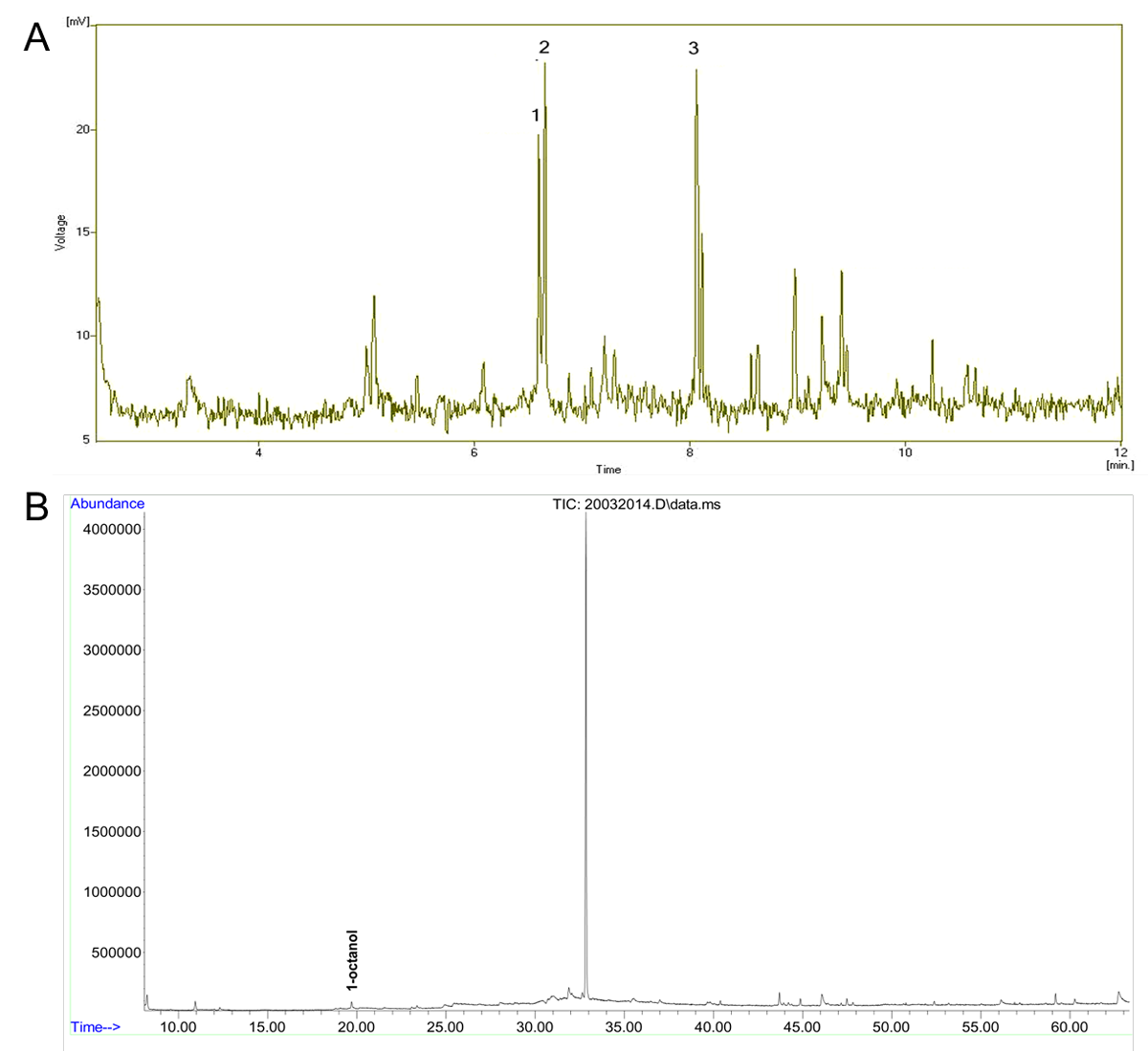

Supplement: S4 Fig — (A) HPLC purified fraction identified as (1) histamine, (2) 4-methyl histamine, and (3) imidazole in tiger moth secretions by GC-MS equipped with a fused silica column. (B) GC-MS analysis identified major volatile compound as 2-methyl-5-propan-2-ylcyclohexa-2,5-diene-1,4-dione (32.8 min) in just emerged moths. The presence of octan-1-ol is detected at 19.9 min. The total content of the octan-1-ol was quantified (0.01 to 1.3%) using standard octanol. 1,4-Benzenedicarboxylic acid, dimethyl ester is identified at 43.69 min. The GC column DB-5ms was used for the analysis. Both females and males release body fluids into the ambient, and females produce more quantity of fluids than males due to the bigger size but qualitatively both male and female secretions are same. (TIF) [file pone.0152787.s006.tif]

A


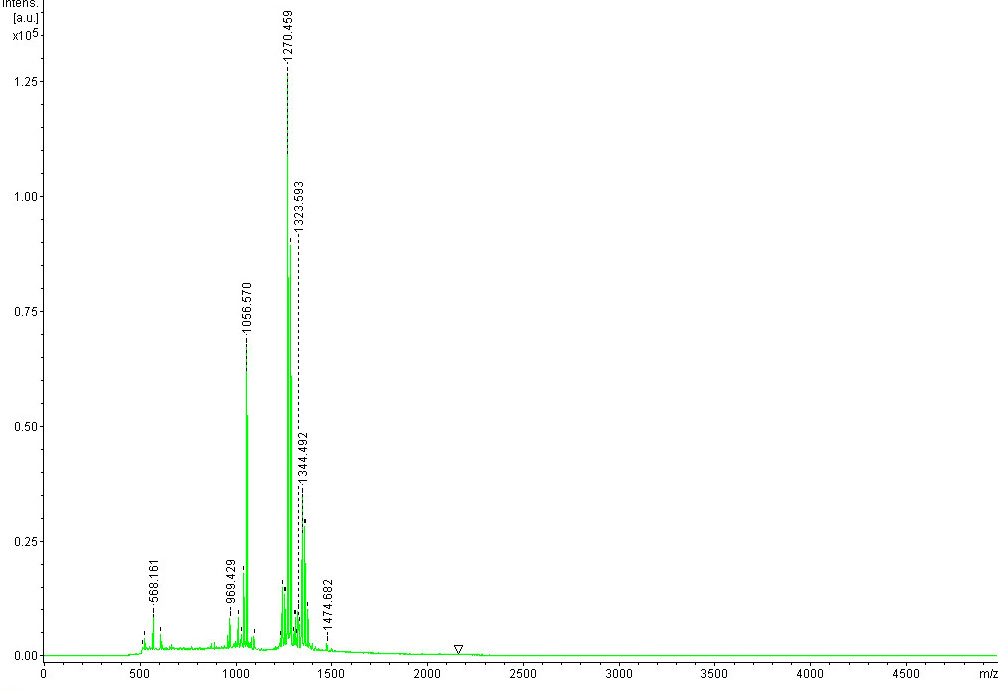


B


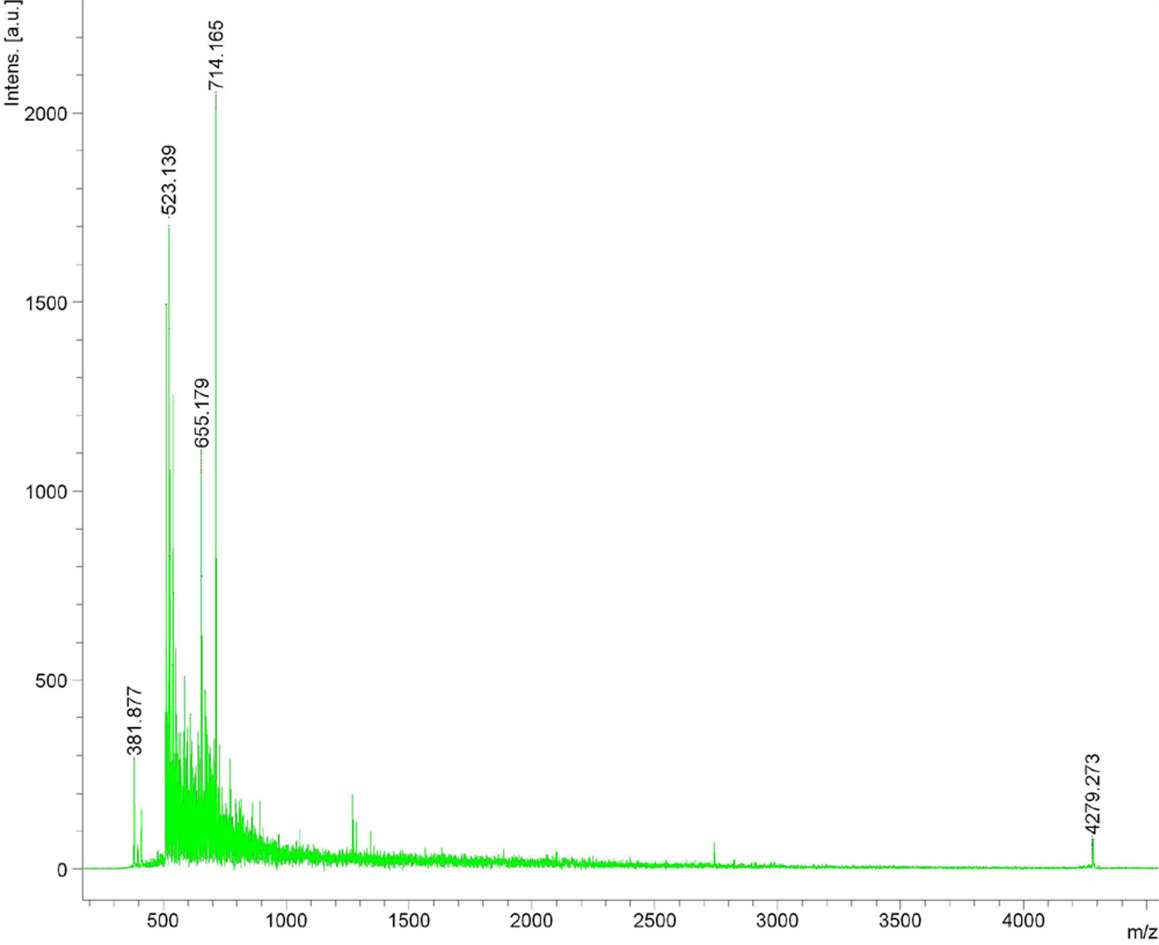

Supplement: S5 Fig — (A) MALDI-TOF-MS analysis demonstrates the presence of peptides (0.5 to 1.4 kDa) in moth secretion. (B) Presence of different peptides including a 4.2 kDa peptide in moth excretion. (DOCX) [file pone.0152787.s007.docx]

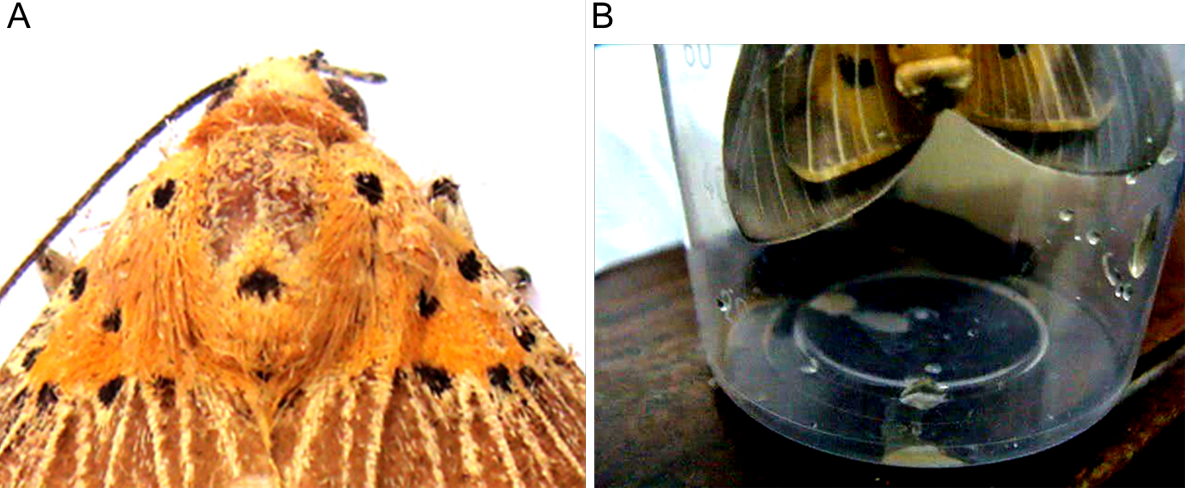

Supplement: S6 Fig — (A) Powdery scales are easily detachable with aging. (B) Cream colored thick fluid is discharged from abdominal and yellow-coloured liquid is discharged from prothoracic points. The photographs were originally taken by P J Wills, corresponding author. We reckoned a maximum of twelve female moths attracted to human habitation in a single occasion with full of detaching scales and hairs. Inhalation of the toxic components from a single moth is enough to cause the disease but cumulative effect of the toxins could generate severity of the disease, sometimes deadly. (TIF) [file pone.0152787.s008.tif]

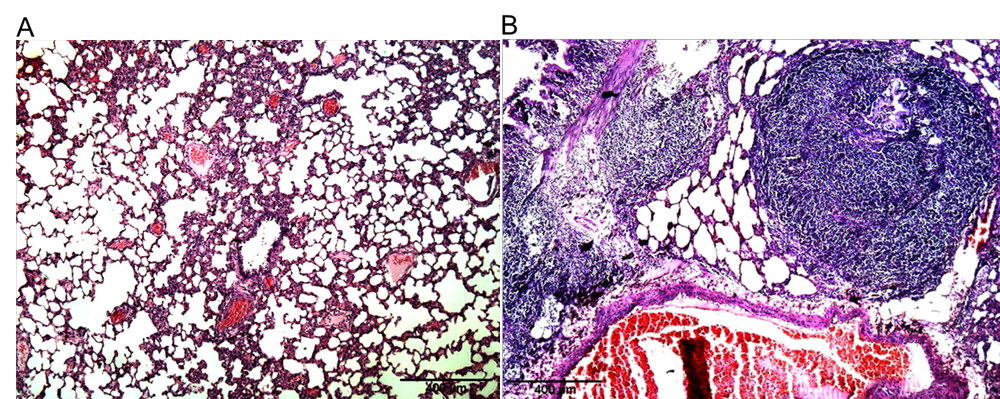

Supplement: S8 Fig — (A) Normal rat lung, (B) Inflamed lung represent extensive perivascular lymphocytic proliferation leading to bronchoconstriction. Low-power view (magnification 40×). Scale bar = = 400 μm. (TIF) [file pone.0152787.s010.tif]

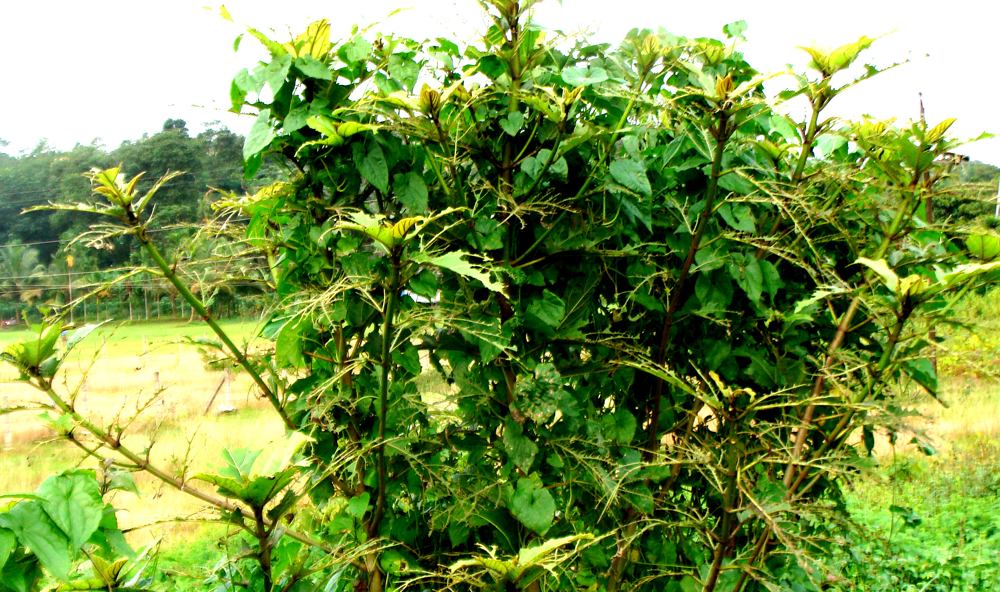

Supplement: S9 Fig — Tiger moth caterpillars select cooler microhabitats during warmest hours to avoid maximum operative temperatures. At high temperatures, female tiger moths prefer to lay eggs on host plants that are covered by twining climbers provide microclimate for the growing instars which function as a perfect shelter from soaring temperatures. With the arrival of monsoon, the caterpillars forage on all level of pioneer habits due to the changes in atmospheric temperature, leading to large scale outbreaks of tiger moths. The photograph was taken by P J Wills. (TIF) [file pone.0152787.s011.tif]
