# Supplementary material for: Population Explosions of Tiger Moth Lead to Lepidopterism Mimicking Infectious Fever Outbreaks
Source: PLoS One. 2016 Apr 13;11(4):e0152787. doi: 10.1371/journal.pone.0152787 (PMC4830441; doi:10.1371/journal.pone.0152787)
Supplement: S1 Table — (DOCX) [file pone.0152787.s014.docx]

S1 Table. Comparison of symptoms (%) among different age groups (yr) during 2007 fever outbreak in Kerala, India.

| **Symptoms/signs** | **Under 30**  **(n = 322)** | **30 to 59**  **(n = 425)** | **60 and above**  **(n = 115)** |
| --- | --- | --- | --- |
| Arthralgia & Myalgia | 90.7 | 98.1 | 100.0 |
| Fever with chills | 85.7 | 89.4 | 93.0 |
| Headache | 77.0 | 79.8 | 75.7 |
| Drowsiness | 82.6 | 91.5 | 94.8 |
| Nausea | 13.0 | 11.1 | 10.4 |
| Vomiting* | 41.6 | 23.1 | 17.4 |
| Edema* | 40.7 | 64.9 | 76.5 |
| Anemia | 30.1 | 24.2 | 21.7 |
| Itching | 38.5 | 44.2 | 53.9 |
| Erythematous rash | 26.7 | 23.8 | 26.1 |
| Hyperpigmentation* | 11.5 | 27.3 | 29.6 |
| Hematoma* | 1.9 | 2.6 | 27.0 |
| Hair loss* | 29.8 | 43.1 | 43.5 |
| Prolonged effect* | 29.5 | 59.8 | 80.9 |

*Considered significant at 0.05 level (Chi-square test).
