# Supplementary material for: Population Explosions of Tiger Moth Lead to Lepidopterism Mimicking Infectious Fever Outbreaks
Source: PLoS One. 2016 Apr 13;11(4):e0152787. doi: 10.1371/journal.pone.0152787 (PMC4830441; doi:10.1371/journal.pone.0152787)
Supplement: S2 Table — (DOCX) [file pone.0152787.s015.docx]

S2 Table. Diagnostic tests/platform used to screen different infectious fever diseases at MIMS.

| **Sl.No.** | **Tests** | **Test Method** | **Company** |
| --- | --- | --- | --- |
| 1 | Chikungunya IgM | Card ICT | CTK Biotech |
| 2 | Dengue IgM | EIA | Panbio |
| 3 | Leptospira IgM | EIA | Panbio |
| 4 | Typhoid | Agglutination/Blood Culture | Span |
| 5 | Malaria | Card ICT/Smear examination | Tulip |
